# eCROPS-CA: a systematic approach toward effective and sustainable cancer prevention in rural China

**DOI:** 10.1186/s12885-015-1253-6

**Published:** 2015-04-08

**Authors:** Jing Chai, Xingrong Shen, Rui Feng, Jing Cheng, Yeji Chen, Zhengqiu Zha, Shangchun Jia, Han Liang, Ting Zhao, Rui Sha, Yong Shi, Kaichun Li, Debin Wang

**Affiliations:** 1 School of Health Service Management, Anhui Medical University, Hefei, Anhui China; 2 Department of Literature Review and Analysis, Library of Anhui Medical University, Hefei, Anhui China; 3 Anhui Center for Disease Control and Prevention, Hefei, Anhui China; 4 Luan Center for Disease Control and Prevention, Luan, Anhui China; 5 Collaboration Center for Cancer Control, First Affiliated Hospital of Anhui Medical University, Hefei, Anhui China

**Keywords:** Cancer, Behavior intervention, Rural communities, Randomized controlled trail, Service integration

## Abstract

**Background:**

Effective prevention against cancers depends heavily on sustained individual efforts practicing protective behaviors and avoiding risk factors in a complex sociocultural context, which requires continuous and personalized supports. Contemporary prevention relies primarily on strategies targeting general population with limited attention being paid to individualized approaches. This study tests a novel package called, in acronym of core intervention components, eCROPS-CA that leverages protective behaviors against over 80% leading cancers among high risk individuals via continuous and tailored counseling by village doctors.

**Methods/Design:**

The study utilizes a quesi-RCT design involving 4320 high risk individuals selected, via rapid and detailed risk assessments, from about 72,000 farmers aged 35+ in 36 administrative villages randomized into equal intervention and delayed intervention arms. The intervention arm receives baseline and semiannual follow up evaluations plus eCROPS-CA for 5 years; while the control arm, only the baseline and follow-up evaluations for the first 5 years and eCROPS-CA starting from the 6^th^ year if the intervention is proved effective. eCROPS-CA comprises electronic supports and supervision (e), counseling cancer prevention (C), recipe for objective behaviors (R), operational toolkit (O), performance-based incentives (P), and screening and assessment (S). Evaluation measures include: incidence and stage of the leading cancers, cancer-related knowledge, attitudes and practices; easy biophysical indicators (e.g., body mass index, blood pressure); intervention compliance, acceptance of the package.

**Discussion:**

The prevention package incorporates key success factors in a synergetic way toward cost-effectiveness and long-term sustainability. It targets a set rather than any single cancer; choses village doctors as key solution to the widespread lack of professional manpower in implementing personalized and thus relatively sophisticated prevention; adopts real-time monitoring in reaching continuous improvement; utilizes smart web aids to enable prioritizing complex determinants of objective behaviors, linking counseling sessions happened at different time points and hence delivering highly coordinated prevention; uses 2-stage risk assessment models in identifying high risk individuals so as to focus on the most needed; applies standardized operation procedures in simplifying and smoothing behavior intervention yet ensuring delivery of essential steps and key elements.

**Trials registry:**

ISRCTN33269053

**Electronic supplementary material:**

The online version of this article (doi:10.1186/s12885-015-1253-6) contains supplementary material, which is available to authorized users.

## Background

Cancers have long been a major cause of human death and diseases-adjusted life year (DAILY) loss [1]. In 2000, new cancer cases and deaths accounted for 10.1 and 6.2 million respectively worldwide. The same figures increased to 10.86 and 6.73 million by 2002 [2]. In 2005, over 7.6 million people died to cancer accounting for 13% of total deaths [3]. Predicted cases and deaths will reach about 15 and 10 million by 2020 [4]. The most common cancers in terms of incidence rate were lung cancer, breast cancer and colorectal cancer and, in terms of mortality rate, lung cancer, stomach cancer and liver cancer [5]. The situation of cancer in China is also most serious. According to China national cancer registration statistics in 2009, standardized cancer incidence rate was 191.72 per 100 thousand and mortality rate, 115.65 [6]. Cumulative incidence rate (age 0–74) in China added up to 21.99% and total incidence cases, over 2.2 million a year [7]. WHO data showed that malignant tumors worldwide took up 5% of total burden caused by all diseases in 2005 [8]. More recent investigations revealed that cancers were the first death cause in cities [9]. In 2006, direct and indirect economic loss due to the disease was 11.32 and 6.00 billion USD respectively representing 4.67% of total medical cost [10].

The harms of cancers to human health can be attributed to prevention, diagnosis and treatment defects. Accordingly, control strategies against the diseases divide into proactive prevention, early detection and appropriate treatment. Tremendous efforts have been invested on clinical diagnosis and treatment for a long time, but associated survival prolongation is rather limited [11-16]. As a result, prevention and early detection are gaining recognition. However, contemporary approaches focus primarily on public education (e.g., disseminating prevention information via various mass media) [17-25], screening service, and drug prevention (e.g., use of folic acid or tamoxifen) and treatment of precancerous conditions (e.g., polyps, Helicobacter pylori infection) [26-33]. Public education is most cost-effective for communicating general knowledge, but its benefit is restricted largely by the fact that there exists a huge disparity between knowledge and behavior [27,28]. Similarly, although a large number of researches have revealed that screening for high-risk groups and some drugs and treatment prevention are highly cost-effective under research conditions, these measures are seldom in use in routine practices of cancer prevention and control [28]; even used, the effectiveness often turned out to be far less from expected [34-40].

Although the huge gap between the actual application of proven preventions and expectations may be attributed to a variety of reasons, lacking personalized behavior promotion may have plaid an important role [41]. Most cancer prevention measures can only work when the target individuals themselves take action. For example, cancer prevention knowledge communicated via mass media needs to be understood, accepted and finally translated into action by intended audiences. Likewise, screening for cancers regularly, treating precancerous lesions and others, all depend heavily on the acceptance and cooperation of the individuals in need of these services. General or non-tailored education and service promotion often fails in initiating or maintaining desired prevention practices due to extreme complexity of the factor system determining these behaviors. On the one hand, the nexus of complex factors makes it hard for ordinary residents to perceive true relationships between prevention measures and cancer incidence and harms. This greatly weakens the motivation for implementing the measures. On the other, effectively changing the outcomes of a complicated behavior determinant system requires integrating multiple measures in a synergetic way. This is to the disadvantage of general “education” and often beyond the ability of ordinary people especially old rural farmers with over 61.8% of illiteracy [42]. Similar challenges also perpetuate diabetes-related behavior intervention. In order to promote sustainable lifestyle modifications preventing progression from pre-diabetes to diabetes, we developed an intervention package called eCROPS and tested it in rural Luan, Anhui, China with encouraging early findings [43]. This study tires to adapt eCROPS to suit cancer behavior intervention and evaluate its efficacy. This new intervention package (further referred to as eCROPS-CA) targets at multi-types of cancers at the same time and stresses reliance on village doctors, integration of cancer prevention with routine medical service, incorporation of multiple strategies in motivating the involved, theory-guided standardization and assessment-based tailoring, and low cost and sustainability.

Although there are a few articles documenting behavior intervention against single type cancer using simple measures (e.g., application of telephone or emails in containing tobacco and alcohol consumption after intestinal polyps resection [44-46]), researches attempting cancer prevention in a systematic way like this are limited. China has a long history of separated systems for disease treatment (medical system) and prevention (CDC system) [47]. The CDC system is founded by the government and launches free cancer preventions (primarily mass media education and case registry); while the medical system depends almost solely on fee for service and provides cancer screening, diagnosis and treatment at patients’ expenses. Although village doctors belong to the medical system, they are not qualified to deliver neither cancer screening nor diagnosis and treatment. So they seldom involve in cancer-related services. The large number of village doctors (over one million) provides enormous potential for reaching far and wide [48] and the historical, social and cultural role of them provides necessary professional legitimacy. Chinese people hold strong beliefs that doctors (including village doctors) are respectful and trustworthy because they save lives and that human body is too complex for ordinary people to fully understand and patients should take their doctors’ advice since they are educated and experienced in dealing with complex health problems [49]. Village doctors enjoy easy access to farmers and knew the patients’ language and level of understanding; while their professional knowledge and experience can greatly facilitate their involvement in cancer intervention and a minimal training can produce highly effective cancer interventionists. More importantly, most cancer prevention service can be arranged to coincide routine medical care which provides a unique teaching moment when health is valued and help from doctors is most needed [50].

## Methods/Design

### Study design

The study utilizes, after one year and a half piloting, a quesi-RCT (randomized controlled trial) design and involves 4320 high risk individuals selected, via rapid risk assessments (RRA) and detailed risk assessments (DRA), from about 72,000 farmers aged 35 or older in 36 administrative villages randomized into equal intervention and delayed intervention arms. The intervention arm receives baseline and semiannual follow up evaluations plus eCROPS-CA for 5 years; while the control arm, only the same baseline and follow up evaluations for the first 5 years and eCROPS-CA starting from year 6 if the intervention is proved effective.

### Study aim

The study aims at demonstrating that eCROPS-CA is effective in preventing leading cancers and high risk individuals in the intervention arm will, compared to those in the delayed intervention condition, show a lower incidence of cancers, improved cancer-related KAP (knowledge, attitudes and practices) and easy-biophysical indicators, and increased use of cancer prevention service. A secondary objective is to establish a sustainable mechanism, in which participating village doctors maintain continuous momentum integrating cancer prevention with routine medical service ever since initiation of this project in resource-poor rural China.

### Study sample

#### Selection and randomization of villages

**Figure 1:**
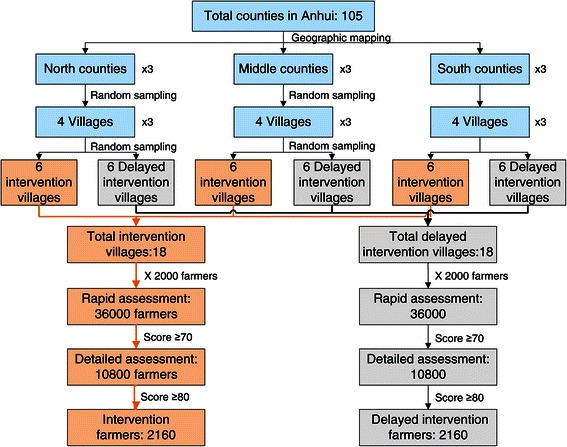
**Study subject sampling and randomization.**

The study involves 36 administrative villages sampled through 3 stages (Figure 1). Stage 1 classifies all the counties in Anhui province into southern, northern and middle areas. Stage 2 randomly selects 3 counties from each of the areas. Stage 3 draws 4 villages with the largest number of famers from each of the counties and randomizes them into two equal groups (i.e., 18 villages in either the intervention or the delayed intervention arms). All delayed intervention villages within half-day walking distance from any intervention one are re-sampled for avoiding cross contamination. The randomizing proceeds in two steps: each of the 9 county CDCs (centers for disease control and prevention) provides a roster and map of administrate villages with over 4000 resident farmers within its jurisdiction; the eCROPS-CA Technique Group from Anhui Medical University performs randomized selection and grouping.

#### Eligibility criteria of participants

The study recruits both village doctors and their patients. All the accredited village doctors working for the clinics of the selected villages are encouraged to participate. Inclusion criteria of patients include men and women who: are 35 years or older; live in the selected villages for over 6 months per year (a large part of rural farmers migrate to cities for temporary jobs every year); meet the cut point score of RRA (≥ the 70th percentile of RRA score) and DRA (≥ the 80^th^ percentile of DRA score). Farmers who have already diagnosed with cancer(s) or mental illness or serious illness or disability are excluded.

#### Sample size

The study sample size derives from the key assumption expected to check that eCROPS-CA may prevent the leading cancers including stomach, trachea/bronchus/lung, esophagus, liver, colon/rectum/anus, breast, cervix, pancreas, and nasopharynx cancers. Based on our previous research, for a village with over 4000 farmers, about 2000 aged 35+ seek service from the village clinic within one year. The rapid assessment identifies 30% of them (i.e., 600) as tentative risk famers (TRFs) and the detailed assessment in turn, 20% of the TRFs as final risk famers (FRFs). In this way, about 72000 famers from the 36 villages will receive RRA, 21600 will go a step further into DRA and a total of 4320, into intervention and control groups. Our previous small scale investigation observed a total of 34 cases of the 9-cancers out of 296 FRFs in 3 years (872 person-years in total). So the annual incidence rate and the 3 year cumulative incidence rate were 3.90% and 12.20% separately. Given these and suppose: intervention has a moderate effect (20% reduction) in the cumulative incidence rate of the 9-cancers in 5 years; significance level (alpha) is set at 0.05 to avoid a Type I error, and power (1–beta), at 95% to avoid a Type II error; c) 5-year follow up attrition is 20%. Therefore, 4320 FRFs should have over 95% chance of detecting significant difference in 5-year cumulative incidence rate of the cancers.

#### Intervention

##### Over view of intervention content and flow

**Figure 2:**
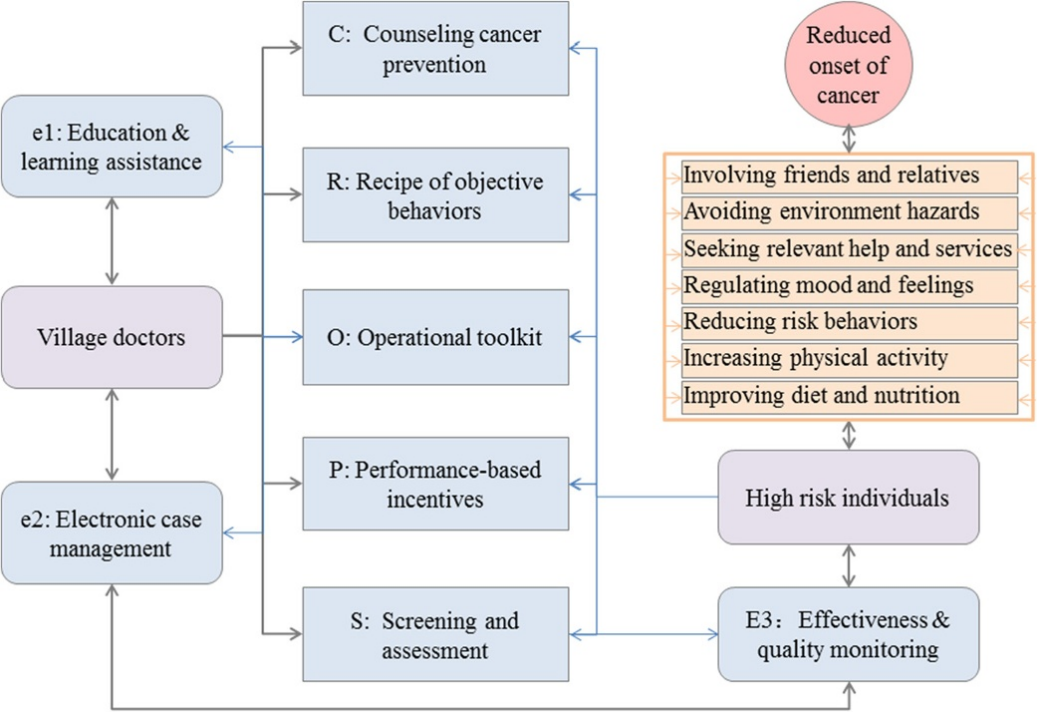
**Intervention components and their relationships.**

**Figure 3:**
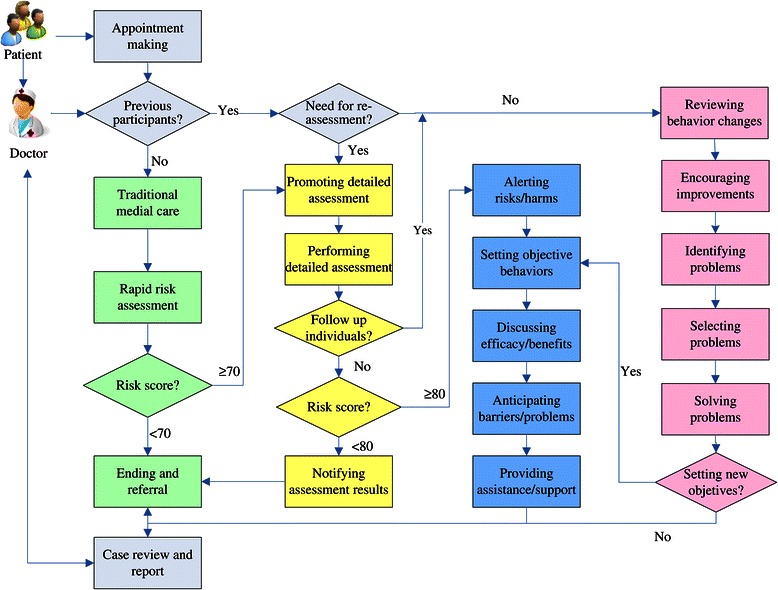
**Logic flow of designed cancer prevention service.**

In addition to existing curative and preventive services, the intervention arm implements eCROPS-CA, where CA stands for cancer while letters in the acronym eCROPS, electronic supports and supervision (e), counseling behavior prevention (C), recipe for objective behaviors (R), operational toolkit (O), performance-based incentives (P), screening and assessment (S) respectively. These components are subject to continuous refinement in accordance with the findings from the semiannual evaluations to be described later especially feedbacks from participating village doctors and farmers. Figure 2 summarizes the components of the intervention package and their relationships. Figure 3 depicts the main procedures of the intervention, the logic flows among these procedures and how they fit with traditional medical service at village clinics. While the following subsections provides item-by-item briefings of eCROPS-CA components.

##### Counseling cancer prevention (C)

Counseling behavior prevention (C) divides into 3 categories, i.e., risk assessment promotion, objective behavior initiation and objective behavior reinforcement. Risk assessment promotion applies to farmers scored higher than the 70^th^ percentile score of RRA and aims at promoting acceptance of DRA. Counseling behavior initiation happens to farmers who have scored higher than the 80^th^ percentile score of the DRA yet not experienced the project-designed behavior intervention and tries to raise their awareness of elevated cancer risks and urges them to take action reducing the risks. It proceeds in 5 steps (blue rectangles in Figure 3): alerting risks and harms; setting objective behaviors; discussing efficacy and benefits; anticipating barriers and problems; providing assistance and supports. Here, objective behavior refers to desired behaviors (such as eating more vegetables and regular self-examination) conducive to cancer prevention. Counseling behavior reinforcement targets follow-up farmers who have received initial behavior change counseling and focuses on reinforcing behavior improvement and solving problems encountered in implementing the changes. It also consists of 5 steps (pink rectangles in Figure 3): reviewing behavior changes; encouraging improvement; identifying problems; selecting problems; solving problems. All counseling sessions utilizes standard operation procedures (SOPs) to ensure delivery of key elements, though the counselor village doctors are encouraged to make the best use of their own experiences. Development of the SOPs employs similar steps, theories and methods we used in deriving the SOPs for diabetes prevention [51,52].

Behavior counseling happens at a monthly (in the first year), bimonthly (in the second year) and quarterly base (in the remaining years). Such arrangement is based on a number of considerations: the counseling need to cover a total of 7 categories of objective behaviors (Figure 2); a typical village doctor serves about 15 patients a day and has enough spare time to deliver counseling; most village clinics locate within 15 minute walking from the farmers and amid their way for going shopping, sending children to schools etc.; walking to and from village clinics may also be viewed as a way to promote additional physical activities, one of the planned objective behaviors.

##### Recipe for objective behaviors (R)

Recipe of objective behaviors (R) consists of a complete list of the behaviors this study tries to make its target farmers to practice and rules/tips for doing so in forms of education leaflets and webpages. The list comprises 7 categories: improving diet and nutrition (e.g., more vegetables, less cured foods); increasing physical activities (e.g., work, housework and leisure-time activities); reducing risk behaviors (e.g., smoking, unprotected sex); regulating mood and feeling (e.g., depression, anxiety); seeking help and services (e.g., cancer screening, treatment for pre-cancerous conditions); avoiding environmental carcinogens (e.g., benzene, arsenic); involving friends and relatives (e.g., spouse or colleagues in exercises). For each of these objective behaviors, the recipe provides bulleted items telling: why one should practice the behavior; how to initiate and maintain the behavior; what problems may encounter in implementing the behavior; how to solve the problems.

##### Operational toolkit (O)

**Figure 4:**
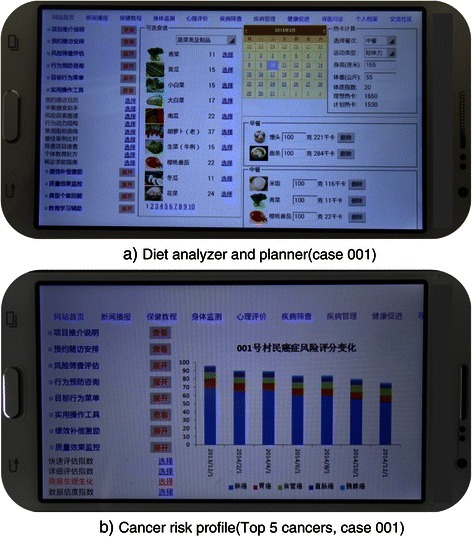
**Sample applications of project operational toolkit viewed using a smart phone.**

Operational toolkit (O) gears with a series of operational tools including: “reference table/lists” of cross-links between cancers and risk factors, contacts of related referral services etc.; “worksheets” for planning diet, physical activities, follow up visits etc.; “easy calculators” for estimating body mass index (BMI), diet calorie intake or glycemic index, activity calorie consumption etc.; and “visual-aids” for demonstrating composition of risk factors and trends in estimated cancer risk score, BMI,blood pressure, plasma glucose etc. All these tools are easily accessible by participating doctors and farmers via the project website at any time (Figure 4).

##### Performance-based incentives (P)

Performance-based incentives (P) aim at generating adequate momentum for village doctors to deliver designed intervention through financial reimbursement, prevention requirement, continuing education credit, and membership of Integrated Service Network. More specifically, participating doctors meeting set performance standard get reimbursed at about $3.5 per case-year; involvement in the cancer prevention satisfies a policy, newly issued by the local health authorities due to the project, mandating that all practicing village doctors must deliver a minimum of prevention service so as to qualify future license renewal; completion of the project training and case reporting and analysis entails awarding of continuing education credits required by annual performance appraisal. In addition, participation in the project automatically gains a membership of the Integrated Service Network that enjoys: free access to periodical circulation of a Newsletter and web-based forum for sharing cases and experiences maintained by members of the Network; technical assistance from higher level network members including members from prefecture and province level hospitals; dual referral privilege within the Network.

##### Screening and assessment (S)

Screening and assessment (S) utilizes a two stage strategy, i.e., RRA (10 to 15 minutes) followed by DRA (20 to 35 minutes), and serves 3-fold purposes: to identify high-risk farmers and thus deliver focused intervention; to inform personalized and outcome-oriented behavior intervention; to enable intervention quality monitoring and supervision. RRA covers all visiting patients aged 35+ who have not received the same assessment in the last two years. It solicits information about risks of developing cancers for individual patients using a web-based structured questionnaire (Additional file 1, Part A) and automatically produces, via the web-based system, a risk score for the patient. If the RRA score is higher than the preset RRA cut point mentioned earlier, a DRA follows which expands the scope and detail of the information collected via the RRA using again a web-based structured instrument (Additional file 1, Part B). DRA also automatically generates a risk score for each patient and if this DRA score is higher than the preset DRA cut point, the patient is eligible to receive further intervention and evaluate on. Calculation of both the RRA and DRA risk scores utilizes a two-stage weighing system (Additional file 1, Part C).

##### Electronic supports and supervision (e)

Electronic supports and supervision (e) adapt from the smart web-aid we developed for preventing diabetes [52] and consists of user-friendly education and learning assistance (ELA, e_1_), high-risk individual management system (HIMS, e_2_), and effectiveness and quality monitoring (EQM, e_3_). The HIMS provides doctors with a whole set of helps ranging from maintaining a dataset of high-risk farmers, scheduling and reminding (via land or cell-phones) follow-up intervention services, reviewing individual performance and intervention history, to facilitating (via built-in standard operating procedures) delivery of intervention counseling etc. The ELA for village doctors comprises: web-based tutorial on implementing the project prevention in both video and textile formats; typical case studies in which participating village doctors are requested to record and post at least 1 bottom case (in terms of compliance with planned behavior changes) bimonthly on a Web-Forum and then experts and other village doctors share experiences coping with similar cases; video and pictorial materials about cancer and its prevention suitable for displaying at village clinics for attending high-risk patients. The EQM assists project managers to: solicit relevant data for monitoring cancer behavior intervention; identify deviation from planned deliverables; and feedback monitoring findings.

#### Control

The control arm maintains existing curative and preventive services without adding any prevention component included in eCROPS-CA except for RRA and DRA.

### Study and data integrity

The study design follows the CONSORT (Consolidated Standards of Reporting Trials) statement [53].

### Measures

The study uses 5 types of outcome and process measures to assess intervention efficacy and sustainability, i.e., diagnosed type and stage of cancers, cancer-related KAP (knowledge, attitudes and practices), easy biophysical indicators, intervention compliance and acceptance of eCROPS-CA (Table 1). In addition, the study also collects related social demographic variables e.g., age, gender, ethnicity, migration patterns, marital status, education.

**Table 1:** **Main outcome measures for assessing intervention efficacy**

Domain of measures	Specific indicators
A) Cases and stages of cancers diagnosed	Cases of the 9 cancers newly diagnosed from last assessment; date and evidence of diagnosis; stage of the cancers at diagnosis.
B) Related knowledge, attitudes and practices	Knowledge about susceptibility and seriousness of cancer; knowledge about effectiveness of practicing each of the objective behaviors; knowledge about benefits of practicing each of the objective behaviors; knowledge about barriers to practicing each of the objective behaviors; knowledge about strategies/techniques for coping with each of the barriers to practicing each of the objective behaviors; practice of each of the objective behaviors.
C) Easy biophysical indicators	Body height; body weight; waist circumstance; hip circumstance; systolic/diastolic blood pressure, fasting plasma glucose etc.
D) Intervention compliance	Proportion of eligible patients who complete detailed risk assessment; proportion of eligible patients who attend at least 10 counseling sessions in the first year of the intervention and at least 5 sessions in each of the following years; proportion of village clinic doctors who complete at least 90% of the counseling sessions assigned.
E) Acceptance of eCROPS-CA	Acceptance, beliefs and comments about the eCROPS-CA as a whole and specific components voiced by key informants, e.g., participating village doctors, farmers and project managers and supervisors.

### Evaluation time points

Evaluation of the intervention package happens at baseline and semiannually thereafter. Each round of field data collection lasts for one week scheduled at the week before doctor training and the last week of the 6^th^, 12^th^, 18^th^, 24^th^, 30^th^, 36^th^, 42^th^, 48^th^, 54^th^, and 60^th^ month after the baseline respectively. Both intervention and control arms receive identical evaluation using same questionnaire, same field data collectors and same assessment time points.

### Data analyses

**Figure 5:**
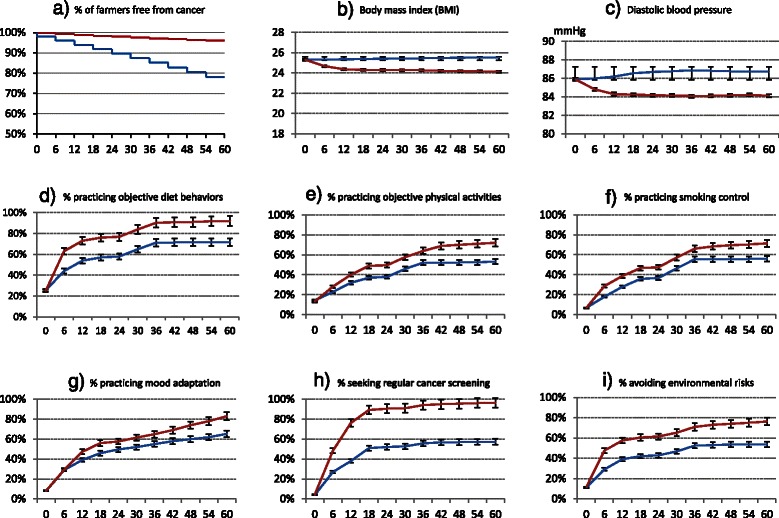
**Sample outcome measures between intervention and control arms (Red lines represent measures for intervention arm and blue lines, delayed intervention arm).**

Analysis of quantitative data proceeds in 3 steps. Initial analysis centers on descriptive summaries intended to examine major deliverables and patterns of the various measurements (Table 1 and Figure 5) and check for normality of the continuous variables. Necessary transformations are explored and selected, if necessary, to induce approximate normality. The next step estimates, using two-sided test of the null hypothesis, the power of differences between the intervention and delayed intervention groups and between different time points in terms of the outcome measures. Considering the possibility that the prevention package may cause earlier cancer diagnosis in the intervention than in the delayed arm and thus results in reduced difference in cancer incidence between the two arms, comparison in composition of cancer stages parallels comparison in cancer incidence rate between the two groups. The last step explores multivariate models, e.g., regression models between: cancer occurrence and various factors assessed via RRA and DRA; objective behaviors and knowledge, attitudes, self-efficacies, prevention services etc.; prevention service compliance and cancer risk scores, age, gender, education, service quality etc. Analysis of qualitative interviews with key informants (e.g., village doctors, farmers, intervention supervisors, project managers) adopts similar perspectives and methods as that used in one of our previous studies [52].

### Ethical considerations

The study involves recruitment, intervention and assessment of patients and village doctors. So it adheres to rigorous human subject protection principles. The study protocol had been reviewed and approved by the Biomedical Ethics Committee of Anhui Medical University (reference number: 20140271). Participation of patients and doctors are voluntary. And written informed consent is sought from all participants.

## Discussion

The cancer prevention package (eCROPS-CA) this study tries to test and refine has several important features. First, eCROPS-CA incorporates key success factors in a synergetic way toward cost-effectiveness and long-term sustainability and targets at a set rather than a single type of cancers. Multiple types of cancers pose problems for interpreting the ultimate outcome (i.e., cancer incidence reduction) of the intervention since, for instance, avoidance of same cases of breast cancer and lung cancer may have different meanings. However, most cancers share similar causes. For example, inadequate intake of vegetables is not only linked with gastric cancer but also colorectal cancer, breast cancer, liver cancer and lung cancer etc. [54,55]. This suggests that increasing vegetable intake may prevent a number of cancers and intervention packages against multiple cancers may prove to be more cost-effective than that focusing on a single cancer. Similarly, comprehensive approaches make it hard to distinguish the effects of specific components within a package and thus selecting and combining elements into a working whole becomes vital yet challenging. In order to properly tackle this challenge, eCROPS-CA adopts soft systems thinking which originates from software development and views all elements in an interactive and holistic way [56]. More specifically, eCROPS-CA first sets an ultimate goal of effective, low cost and sustainable interventions against common cancers and then identifies key success factors for reaching this end, devices necessary intervention components satisfying all these factors, and finally forms a practical whole. This development strategy should ensure that the whole intervention package is goal-oriented and each component included has clear and important contributions to the ultimate goal.

Second, eCROPS-CA adopts real-time effectiveness and quality monitoring in leveraging continuous improvement. Here, “real-time” means that the majority of data required by the monitoring derive from ad hoc records of routine prevention rather than from separate data reporting or collection. For example, during counseling sessions, the smart web aid always proposes step-wise topics for discussion, each of which follows a checkbox list that enables the counselor doctor to record essential items about what the counselee patient has already know or done. And only when the doctor has clicked all the required checkboxes, can he/she proceed with the next step. This real-time recoding not only saves costs but also enhances accuracy of data collection. More importantly, although managerial monitoring relies heavily on real-time data, the primary purpose of all these recordings is to help the village doctors to improve their prevention service and performance. Taking the above example again, the checkboxes are designed mainly for branching and informing next step counseling rather than managerial monitoring. This reduces potential conflict of interests that perpetuates most self-report-based project monitoring since the benefits (e.g., better tailored service, earlier detection and correction of weaknesses) over-weight the dis-benefits (e.g., temporary or partial reduction in performance assessment) for maintaining high data collection fidelity. In addition, the monitoring stresses easy biophysical indicators (e.g., BMI, blood pressure, plasma glucose) which incur almost no pain and the least cost for both service providers and receivers and all of these indicators are closely linked to the health of high risk individuals on the one hand and the performance of the village doctors on the other. This clear relationship makes the monitoring most convincing for all stakeholders.

Third, eCROPS-CA utilizes powerful recording, retrieving and processing abilities of computer systems to enable prioritizing complex determinants of objective behaviors, linking counseling sessions happened at different time points and hence delivering highly coordinated prevention services. Although computers and internet are available at most village clinics in Anhui and throughout China and most village doctors have elementary computer literacy [57], there are considerable fears about the ability of village doctors in rural China to use computerized systems. Our preliminary researches suggest that electronic applications are both acceptable and manageable to doctors even in resource-poor areas [43,52]. Given the user-friendly education and learning assistance, all the village doctors learned to use the system at a one day orientation workshop. Then, after 2 to 4 weeks of self-practice, encouragement, and problem inquiring and answering, most village doctors became confident users. By examining this protocol, one can easily find that eCROPS-CA is almost infeasible without the “e” component. If proved effective, this study should inform leveraging electronic supports in exploring new cancer prevention models and techniques.

Other characteristics of eCROPS-CA include: it uses stage-wise risk assessment models in identifying high risk individuals so as to greatly narrow down the scale of intervention and focus scarce resources on the most needed; it applies standardized operation procedures (SOPs) derived from proven theories (e.g., health belief model, motivational interviewing) and best practices in simplifying and smoothing behavior intervention yet ensuring delivery of essential steps and key success elements; it employs consecutive assessment and feedbacks in tailoring interventions to individual’s dynamic risk status and influence factors. These will be further addressed separately.

## Additional file

Additional file 1:
**Detailed risk assessment instrument and scoring systems.**
