# Supplementary material for: eCROPS-CA: a systematic approach toward effective and sustainable cancer prevention in rural China
Source: BMC Cancer. 2015 Apr 8;15:233. doi: 10.1186/s12885-015-1253-6 (PMC4416351; doi:10.1186/s12885-015-1253-6)
Supplement: Additional file 1: — Detailed risk assessment instrument and scoring systems. [file 12885_2015_1253_MOESM1_ESM.docx]

**Additional file 1: Detailed risk assessment instrument and scoring systems**

1. **Rapid risk assessment instrument and value assignment**

**Re. number: _________; Gender: □Male; □Female; Birthdate: ___DD___MM_____YYYY.**

**Do you often have any of the following digestive** **symptoms?**

- Toothache[X1=1 if checked or 0 if unchecked]
- Food reflux [X2=1 if checked or 0 if unchecked]
- Abdominal discomfort[X3=1 if checked or 0 if unchecked]
- Stomachache[X4=1 if checked or 0 if unchecked]
- Constipation[X5=1 if checked or 0 if unchecked]
- Stool with blood and mucus[X6=1 if checked or 0 if unchecked]
- Appetite decreases[X7=1 if checked or 0 if unchecked]
- Hepatalgia[X8=1 if checked or 0 if unchecked]
- Skin and sclera icterus[X9=1 if checked or 0 if unchecked]

**Have you ever been diagnosed with any of following digestive diseases?**

- Tooth loss or cavities[X10=1 if checked or 0 if unchecked]
- Chronic gastritis[X11=1 if checked or 0 if unchecked]
- Chronic gastritis ulcer[X12=1 if checked or 0 if unchecked]
- Helicobacter pyloriInfection[X13=1 if checked or 0 if unchecked]
- Duodenal ulcer[X14=1 if checked or 0 if unchecked]
- Hepatitis[X15=1 if checked or 0 if unchecked]
- Cirrhosis[X16=1 if checked or 0 if unchecked]
- Fatty liver[X17=1 if checked or 0 if unchecked]
- Cholecystitis /Cholelithiasis[X18=1 if checked or 0 if unchecked]
- Chronic appendicitis /enteritis[X19=1 if checked or 0 if unchecked]
- Intestinal polyps [X20=1 if checked or 0 if unchecked]
- Pancreatitis [X21=1 if checked or 0 if unchecked]
- Hemorrhoids [X22=1 if checked or 0 if unchecked]

**Do you often have any of the following respiratory** **symptoms?**

- Chest distress/dyspnea[X23=1 if checked or 0 if unchecked]
- Chest pain[X24=1 if checked or 0 if unchecked]
- Cough [X25=1 if checked or 0 if unchecked]
- Rhinobyon[X26=1 if checked or 0 if unchecked]
- Running nose[X27=1 if checked or 0 if unchecked]

**Have you been diagnosed with following respiratory disease?**

- Tuberculosis [X28=1 if checked or 0 if unchecked]
- Chronic obstructive pulmonary disease [X29=1 if checked or 0 if unchecked]
- Asthma [X30=1 if checked or 0 if unchecked]
- Pneumonia [X31=1 if checked or 0 if unchecked]
- Bronchitis [X32=1 if checked or 0 if unchecked]
- Emphysema [X33=1 if checked or 0 if unchecked]
- Silicosis [X34=1 if checked or 0 if unchecked]
- Pneumoconiosis [X35=1 if checked or 0 if unchecked]
- Chronic rhinitis [X36=1 if checked or 0 if unchecked]
- Epstein-Barr virus infection [X37=1 if checked or 0 if unchecked]

**Do you often have any of the following** **reproductive symptoms (applies only to females)?**

- Breast pain[X38=1 if checked or 0 if unchecked]
- Breast mass[X39=1 if checked or 0 if unchecked]
- Nipple discharge[X40=1 if checked or 0 if unchecked]
- Colporrhagia[X41=1 if checked or 0 if unchecked]
- Abnormal leucorrhea [X42=1 if checked or 0 if unchecked]
- Pelvic mass[X43=1 if checked or 0 if unchecked]
- Hypogastralgia[X44=1 if checked or 0 if unchecked]
- Lumbago pain and tenesmus[X45=1 if checked or 0 if unchecked]

**Have you ever been diagnosed with any of following reproductive diseases(applies only to females)?**

- Mastitis[X46=1 if checked or 0 if unchecked]
- Hyperplasia of mammary glands[X47=1 if checked or 0 if unchecked]
- Fibroadenoma of breast[X48=1 if checked or 0 if unchecked]
- Galactoma[X49=1 if checked or 0 if unchecked]
- Gynecological inflammation（cervicitis/ vaginitis/ pelvic inflammatory/ endometritis）[X50=1 if checked or 0 if unchecked]
- STIs (condyloma acuminatum/ syphilis/ genital herpes/ gonorrhea/HIV) [X51=1 if checked or 0 if unchecked]
- HPV (human papillomavirus) infection[X52=1 if checked or 0 if unchecked]
- Irregular menstruation/ Dysmenorrhea[X53=1 if checked or 0 if unchecked]
- Sterility [X54=1 if checked or 0 if unchecked]
- Uterine fibroid[X55=1 if checked or 0 if unchecked]
- Cervical cyst[X56=1 if checked or 0 if unchecked]
- Ovarian cyst[X57=1 if checked or 0 if unchecked]
- Postmenopausal hormone receptor positive[X58=1 if checked or 0 if unchecked]

**Do you have any of the following general disease or symptom?**

- Diabetes [X59=1 if checked or 0 if unchecked]
- Hypertension [X60=1 if checked or 0 if unchecked]
- Hyperlipidemia [X61=1 if checked or 0 if unchecked]
- Rheumatoid arthritis [X62=1 if checked or 0 if unchecked]
- Systemic lupus erythematosus[X63=1 if checked or 0 if unchecked]
- Insomnia/ dreaminess[X64=1 if checked or 0 if unchecked]
- Blush after drinking[X65=1 if checked or 0 if unchecked]
- Overweight/obesity[X66=1 if checked or 0 if unchecked]
- Underweight[X67=1 if checked or 0 if unchecked]

**Have you ever received any of the following treatment?**

- Laxatives [X68=1 if checked or 0 if unchecked]
- Vitamin B12 [X69=1 if checked or 0 if unchecked]
- Cholecystectomy [X70=1 if checked or 0 if unchecked]
- Gastrectomy[X71=1 if checked or 0 if unchecked]
- Calcium channel blocker (Nifedipine/Verapamil/Diltiazem) [X72=1 if checked or 0 if unchecked]
- Estrogen[X73=1 if checked or 0 if unchecked]

**Please offer the following information (applies only to females)?**

- Age of first menstruation_____[X74=1 if the entered “age” is less than 13 ,else 0]
- Days of menstruation per time_____[X75=1 if the entered “days” is over 3 days ,else 0]
- Age of menopause_____[X76=1 if the entered “age” is more than 50, else 0]
- Age of first marriage_____[X77=1 if the entered “age” is less than 20, else 0]
- Times of marriage_____[X78=1 if the entered “times” is more than 1,else 0]
- Age of first sexual activity_____[X79=1 if the entered “age” is less than 16, else 0]
- Age of first pregnancy_____[X80=1 if the entered “age” is less than 20, else 0]
- Age of first parturition_____[X81=1 if the entered “age” is less than 20, else 0]
- Times of parturition_____[X82=1 if the entered “times” is more than 2,else 0]
- Times of abortions_____[X83=1 if the entered “times” is more than 3,else 0]
- Accumulative years of taking contraceptives_____[X84=1 if the entered “years” is more than 10,else 0]
- Accumulative months of breast feeding_____[X85=1 if the entered “months” is less than 6,else 0]
- Times of premature birth_____[X86=1 if the entered “times” is more than 1,else 0]

**Have any of your following relatives been diagnosed with any kind of cancers?**

- Parent[V1=1.5 if checked or 0 if unchecked]
- Brother/Sister[V2=1 if checked or 0 if unchecked]
- Child[V3=1.5 if checked or 0 if unchecked]
- Grand Parent[V4=1 if checked or 0 if unchecked]
- Uncle/Aunt[V5=1 if checked or 0 if unchecked]
- cousin[V6=1 if checked or 0 if unchecked]
- Nephew/niece [V7=1 if checked or 0 if unchecked]

[X87=(V1+V2+V3+V4+V5+V6+V7)/4]

**Have any of your relatives been diagnosed with the following condition(s)?**

- Diabetes[X88=1 if checked or 0 if unchecked]
- Hepatitis[X89=1 if checked or 0 if unchecked]
- Epstein-Barr virus infection[X90=1 if checked or 0 if unchecked]

**Have any of your long-term partner(s) been diagnosed with urogenital system inflammation?**

- Yes[X91=1]
- No[X91=0]

**Do you often eat the following food(s)?**

- Pickled food [X92=1 if checked or 0 if unchecked]
- Dried food[X93=1 if checked or 0 if unchecked]
- Smoked food[X94=1 if checked or 0 if unchecked]
- Fried food[X95=1 if checked or 0 if unchecked]
- Spicy food[X96=1 if checked or 0 if unchecked]
- Processed food[X97=1 if checked or 0 if unchecked]
- Hard food (e.g., rice crust) [X98=1 if checked or 0 if unchecked]
- Fermented food[X99=1 if checked or 0 if unchecked]
- Leftovers[X100=1 if checked or 0 if unchecked]
- Cottonseed oil [X101=1 if checked or 0 if unchecked]

**Do you have any of the following diet preference(s)?**

- High salt food[X102=1 if checked or 0 if unchecked]
- Fat/oil-rich food[X103=1 if checked or 0 if unchecked]
- Meat dominated meals[X104=1 if checked or 0 if unchecked]

**Do you have the following dietary habit(s)?**

- Eating too full[X105=1 if checked or 0 if unchecked]
- Eating fast[X106=1 if checked or 0 if unchecked]
- Eating hot food[X107=1 if checked or 0 if unchecked]
- Eating cold food[X108=1 if checked or 0 if unchecked]
- Eating at irregular time[X109=1 if checked or 0 if unchecked]
- Eating within 1 hour before sleep[X110=1 if checked or 0 if unchecked]
- Drinking tea[X111=0 if checked or 1 if unchecked]
- Drinking coffee[X112=1 if checked or 0 if unchecked]
- Drinking alcohol[X113=1 if checked or 0 if unchecked]

**Which of following describes you?**

- Smoker[X114=1 if checked or 0 if unchecked]
- Sedentary person [X115=1 if checked or 0 if unchecked]
- Night owl[X116=1 if checked or 0 if unchecked]

**Have you ever taken the following job?**

- Renovation worker/painter[X117=1 if checked or 0 if unchecked]
- Carpenter[X118=1 if checked or 0 if unchecked]
- Cooker[X119=1 if checked or 0 if unchecked]
- Barber[X120=1 if checked or 0 if unchecked]
- Aluminum worker[X121=1 if checked or 0 if unchecked]
- Welder[X122=1 if checked or 0 if unchecked]
- Miner[X123=1 if checked or 0 if unchecked]
- Chemical worker[X124=1 if checked or 0 if unchecked]
- Boiler worker[X125=1 if checked or 0 if unchecked]
- Asphalt/resin worker[X126=1 if checked or 0 if unchecked]
- Dye/pigments worker[X127=1 if checked or 0 if unchecked]
- Rubber/plastic worker[X128=1 if checked or 0 if unchecked]
- Gas worker[X129=1 if checked or 0 if unchecked]
- X-ray or radiation related worker[X130=1 if checked or 0 if unchecked]

**Have you been exposed any of the following harmful materials for a long time?**

- Pesticides[X131=1 if checked or 0 if unchecked]
- Cooking fumes[X132=1 if checked or 0 if unchecked]
- Firewood smoke[X133=1 if checked or 0 if unchecked]
- Soot[X134=1 if checked or 0 if unchecked]
- Dust or cotton dust[X135=1 if checked or 0 if unchecked]
- Mosquitocide[X136=1 if checked or 0 if unchecked]
- Formaldehyde[X137=1 if checked or 0 if unchecked]
- Coal tar[X138=1 if checked or 0 if unchecked]
- Chlorophenol[X139=1 if checked or 0 if unchecked]
- Arsenic[X140=1 if checked or 0 if unchecked]
- Chrome[X141=1 if checked or 0 if unchecked]
- Radon[X142=1 if checked or 0 if unchecked]
- Asbestos [X143=1 if checked or 0 if unchecked]
- benzene[X144=1 if checked or 0 if unchecked]

**Have you ever lived in the following housing conditions?**

- Newly built/decorated house[X145=1 if checked or 0 if unchecked]
- House located near polluting factories [X146=1 if checked or 0 if unchecked]
- House using open and unprocessed water sources[X147=1 if checked or 0 if unchecked]

**Have you ever experienced the following life events?**

- Death of beloved/family member(s) [X148=1 if checked or 0 if unchecked]
- Major disease/ injury of beloved/family member(s) [X149=1 if checked or 0 if unchecked]
- Major disease/ injury of self [X150=1 if checked or 0 if unchecked]
- Major property damage[X151=1 if checked or 0 if unchecked]
- Long -term conflict/dispute with neighbor(s) [X152=1 if checked or 0 if unchecked]
- Long -term conflict/dispute with colleague(s) [X153=1 if checked or 0 if unchecked]
- Breakup/discord with spouse or boy/girl friend[X154=1 if checked or 0 if unchecked]
- Highly stressful/intensive work[X155=1 if checked or 0 if unchecked]
- Natural/man-caused disaster(s) [X156=1 if checked or 0 if unchecked]
- Litigation(s) [X157=1 if checked or 0 if unchecked]

**Do you often have the following feeling/experience?**

- Impatience or impulse[X158=1 if checked or 0 if unchecked]
- Anxiety[X159=1 if checked or 0 if unchecked]
- Tension/stress[X160=1 if checked or 0 if unchecked]
- Melancholy or repression[X161=1 if checked or 0 if unchecked]
- Depression/frustration[X162=1 if checked or 0 if unchecked]
- Burring things in heart rather than sharing them [X163=1 if checked or 0 if unchecked]
- Sulking[X164=1 if checked or 0 if unchecked]

**B) Detailed risk assessment instrument and value assignment**

**X1: Toothache (if unchecked in rapid assessment, X1= 0 and skip to X2)**

1. How old were you when you started frequent toothache?

- 10 and less [X1a=1.2]
- 1 1-20 [X1a=1.0]
- 21-30 [X1a=0.8]
- 31-40 [X1a=0.6]
- 41-50 [X1a=0.4]
- 51 and older [X1a=0.2]

1. How many times did you have toothache a year?

- 1-5[X1b=0.2]
- 6-10 [X1b=0.4]
- 11-15 [X1b=0.6]
- 16-20 [X1b=0.8]
- 21 -25[X1b=1.0]
- 26 and more[X1b=1.2]

[X1=(X1a+X1b)/2]

**X2: Food reflux (if unchecked in rapid assessment, X2= 0 and skip to X3)**

1. How old were you when you began to feel frequent food reflux?

- 10 and less [X2a=1.2]
- 1 1-20 [X2a=1.0]
- 21-30 [X2a=0.8]
- 31-40 [X2a=0.6]
- 41-50 [X2a=0.4]
- 51 and older [X2a=0.2]

1. How many times did you experience food reflux a year?

- 1-5[X2b=0.2]
- 6-10 [X2b=0.4]
- 11-15 [X2b=0.6]
- 16-20 [X2b=0.8]
- 21 -25[X2b=1.0]
- 26 and more[X2b=1.2]

[X2=(X2a+X2b)/2]

**X3: Abdominal discomfort (if unchecked in rapid assessment, X3= 0 and skip to X4)**

1. How old were you when you began to feel frequent abdominal discomfort?

- 10 and less [X3a=1.2]
- 1 1-20 [X3a=1.0]
- 21-30 [X3a=0.8]
- 31-40 [X3a=0.6]
- 41-50 [X3a=0.4]
- 51 and older [X3a=0.2]

1. How many times did you feel abdominal discomfort a year?

- 1-5[X3b=0.2]
- 6-10 [X3b=0.4]
- 11-15 [X3b=0.6]
- 16-20 [X3b=0.8]
- 21 -25[X3b=1.0]
- 26 and more[X3b=1.2]

[X3=(X3a+X3b)/2]

**X4: Stomachache (if unchecked in rapid assessment, X4= 0 and skip to X5)**

1. How old were you when you began to feel frequent stomachache?

- 10 and less [X4a=1.2]
- 1 1-20 [X4a=1.0]
- 21-30 [X4a=0.8]
- 31-40 [X4a=0.6]
- 41-50 [X4a=0.4]
- 51 and older [X4a=0.2]

1. How many times did you suffer from stomachache a year?

- 1-5[X4b=0.2]
- 6-10 [X4b=0.4]
- 11-15 [X4b=0.6]
- 16-20 [X4b=0.8]
- 21 -25[X4b=1.0]
- 26 and more[X4b=1.2]

[X4=(X4a+X4b)/2]

**X5: Constipation (if unchecked in rapid assessment, X5= 0 and skip to X6)**

1. How old were you when you began to present frequent constipation?

- 10 and less [X5a=1.2]
- 1 1-20 [X5a=1.0]
- 21-30 [X5a=0.8]
- 31-40 [X5a=0.6]
- 41-50 [X5a=0.4]
- 51 and older [X5a=0.2]

1. How many times did you experience constipation a year?

- 1-5[X5b=0.2]
- 6-10 [X5b=0.4]
- 11-15 [X5b=0.6]
- 16-20 [X5b=0.8]
- 21 -25[X5b=1.0]
- 26 and more[X5b=1.2]

[X5=(X5a+X5b)/2]

**X6: Stool with blood/mucus (if unchecked in rapid assessment, X6= 0 and skip to X7)**

1. How old were you when you began to present frequent stool blood and mucus?

- 10 and less [X6a=1.2]
- 1 1-20 [X6a=1.0]
- 21-30 [X6a=0.8]
- 31-40 [X6a=0.6]
- 41-50 [X6a=0.4]
- 51 and older [X6a=0.2]

1. How many times did you present stool blood and mucus a year?

- 1-5[X6b=0.2]
- 6-10 [X6b=0.4]
- 11-15 [X6b=0.6]
- 16-20 [X6b=0.8]
- 21 -25[X6b=1.0]
- 26 and more[X6b=1.2]

[X6=(X6a+X6b)/2]

**X7: Appetite decrease (if unchecked in rapid assessment, X7= 0 and skip to X8)**

1. How old were you when you began to feel frequent anorexia/appetite decreases?

- 10 and less [X7a=1.2]
- 1 1-20 [X7a=1.0]
- 21-30 [X7a=0.8]
- 31-40 [X7a=0.6]
- 41-50 [X7a=0.4]
- 51 and older [X7a=0.2]

1. How many times did you experience anorexia/appetite decreases a month?

- 1-5[X7b=0.2]
- 6-10 [X7b=0.4]
- 11-15 [X7b=0.6]
- 16-20 [X7b=0.8]
- 21 -25[X7b=1.0]
- 26 and more[X7b=1.2]

[X7=(X7a+X7b)/2]

**X8: Hepatalgia (if unchecked in rapid assessment, X8= 0 and skip to X9)**

1. How old were you when you began to feel frequent hepatalgia?

- 10 and less [X8a=1.2]
- 1 1-20 [X8a=1.0]
- 21-30 [X8a=0.8]
- 31-40 [X8a=0.6]
- 41-50 [X8a=0.4]
- 51 and older [X8a=0.2]

1. How many times did you experience hepatalgia a year?

- 1-5[X8b=0.2]
- 6-10 [X8b=0.4]
- 11-15 [X8b=0.6]
- 16-20 [X8b=0.8]
- 21 -25[X8b=1.0]
- 26 and more[X8b=1.2]

[X8=(X8a+X8b)/2]

**X9: Skin and sclera icterus (if unchecked in rapid assessment, X9= 0 and skip to X10)**

1. How old were you when you began to present skin and sclera icterus?

- 10 and less [X9a=1.2]
- 1 1-20 [X9a=1.0]
- 21-30 [X9a=0.8]
- 31-40 [X9a=0.6]
- 41-50 [X9a=0.4]
- 51 and older [X9a=0.2]

1. How many years in total have you experienced skin and sclera icterus?

- 0-1[X9b=0.2]
- 2-3[X9b=0.4]
- 4-5[X9b=0.6]
- 6-7 [X9b=0.8]
- 8-9[X9b=1.0]
- 10 and more[X9b=1.2]

[X9=(X9a+X9b)/2]

**X10: Tooth loss/cavities (if unchecked in rapid assessment, X10= 0 and skip to X11)**

1. How old were you when you first found tooth cavity?

- 10 and less [X10a=1.2]
- 1 1-20 [X10a =1.0]
- 21-30 [X10a =0.8]
- 31-40 [X10a =0.6]
- 41-50 [X10a =0.4]
- 51 and older [X10a =0.2]

1. How many cavity teeth have you had in total?

- 1-2 [X10b=0.2]
- 3-4 [X10b =0.4]
- 5-6 [X10b =0.6]
- 7-8 [X10b =0.8]
- 9-10 [X10b =1.0]
- 11 and more [X10b =1.2]

1. How many teeth have you lost?

- 1-2 [X10c=0.2]
- 3-4 [X10c =0.4]
- 5-6 [X10c =0.6]
- 7-8 [X10c =0.8]
- 9-10 [X10c =1.0]
- 11 and more [X10c =1.2]

[X10=(X10a+X10b+X10c)/3]

**X11: Chronic gastritis (if unchecked in rapid assessment, X11= 0 and skip to X12)**

1. How old were you when you first diagnosed with gastritis?

- 10 and less [X11a=1.2]
- 1 1-20 [X11a =1.0]
- 21-30 [X11a =0.8]
- 31-40 [X11a =0.6]
- 41-50 [X11a =0.4]
- 51 and older [X11a =0.2]

1. How many times in total have you experienced recurrence/relapse of chronic gastritis?

- ____times(X11b=entered times)

1. How many days did it generally last for a recurrence/relapse of chronic gastritis?

- ____days(X11c=entered days)

[X11bc=0.2, 0.4, 0.6, 0.8, 1.0, 1.2 respectively if X11b*X11c=1-180, 181-360, 361-720, 721-1080, 1081-1440, 1441 and over]

[X11=(X11a+X11bc)/2]

**X12: Chronic gastritis ulcer (if unchecked in rapid assessment, X12= 0 and skip to X13)**

1. How old were you when you first diagnosed with chronic gastritis ulcer?

- 10 and less [X12a=1.2]
- 1 1-20 [X12a =1.0]
- 21-30 [X12a =0.8]
- 31-40 [X12a =0.6]
- 41-50 [X12a =0.4]
- 51 and older [X12a =0.2]

1. How many times in total have you had recurrence/relapse of gastritis ulcer?

- ____times(X12b=entered times)

1. How many days did it generally last for a recurrence/relapse of gastritis ulcer?

- ____days(X12c=entered days)

[X12bc=0.2, 0.4, 0.6, 0.8, 1.0, 1.2 respectively if X12b*X12c=1-180, 181-360, 361-720, 721-1080, 1081-1440, 1441 and over]

[X12=(X12a+X12bc)/2]

**X13: Helicobacter pylori infection (if unchecked in rapid assessment, X13= 0 and skip to X14)**

1. How old were you when you first diagnosed with helicobacter pylori infection?

- 10 and less [X13a=1.2]
- 1 1-20 [X13a =1.0]
- 21-30 [X13a =0.8]
- 31-40 [X13a =0.6]
- 41-50 [X13a =0.4]
- 51 and older [X13a =0.2]

1. How many times in total have you had recurrence/relapse of pylori infection?

- ____times(X13b=entered times)

1. How many days did it generally last for a recurrence/relapse of pylori infection?

- ____days(X13c=entered days)

[X13bc=0.2, 0.4, 0.6, 0.8, 1.0, 1.2 respectively if X13b*X13c=1-180, 181-360, 361-720, 721-1080, 1081-1440, 1441 and over]

[X13=(X13a+X13bc)/2]

**X14: Duodenal ulcer (if unchecked in rapid assessment, X14= 0 and skip to X15)**

1. How old were you when first diagnosed with duodenal ulcer?

- 10 and less [X14a=1.2]
- 1 1-20 [X14a =1.0]
- 21-30 [X14a =0.8]
- 31-40 [X14a =0.6]
- 41-50 [X14a =0.4]
- 51 and older [X14a =0.2]

1. How many times in total have you experienced recurrence/relapse of duodenal ulcer?

- ____times(X14b=entered times)

1. How many days did it generally last for a recurrence/relapse of duodenal ulcer?

- ____days(X14c=entered days)

[X14bc=0.2, 0.4, 0.6, 0.8, 1.0, 1.2 respectively if X14b*X14c=1-180, 181-360, 361-720, 721-1080, 1081-1440, 1441 and over]

[X14=(X14a+X14bc)/2]

**X15: Hepatitis (if unchecked in rapid assessment, X15= 0 and skip to X16)**

1. How old were you when you first diagnosed with hepatitis?

- 10 and less [X15a=1.2]
- 1 1-20 [X15a =1.0]
- 21-30 [X15a =0.8]
- 31-40 [X15a =0.6]
- 41-50 [X15a =0.4]
- 51 and older [X15a =0.2]

1. How many times in total have you experienced recurrence/relapse of hepatitis?

- ____times(X15b=entered times)

1. How many days did it generally last for a recurrence/relapse of hepatitis?

- ____days(X15c=entered days)

[X15bc=0.2, 0.4, 0.6, 0.8, 1.0, 1.2 respectively if X15b*X15c=1-180, 181-360, 361-720, 721-1080, 1081-1440, 1441 and over]

[X15=(X15a+X15bc)/2]

**X16: Cirrhosis (if unchecked in rapid assessment, X16= 0 and skip to X17)**

1. How old were you when you first diagnosed with cirrhosis?

- 10 and less [X16=1.2]
- 1 1-20 [X16=1.0]
- 21-30 [X16=0.8]
- 31-40 [X16=0.6]
- 41-50 [X16=0.4]
- 51 and older [X16=0.2]

**X17: Fatty liver (if unchecked in rapid assessment, X17= 0 and skip to X18)**

1. How old were you when you first diagnosed with fatty liver?

- 10 and less [X17=1.2]
- 1 1-20 [X17 =1.0]
- 21-30 [X17 =0.8]
- 31-40 [X17 =0.6]
- 41-50 [X17 =0.4]
- 51 and older [X17 =0.2]

**X18: Cholecystitis/Cholelithiasis (if unchecked in rapid assessment, X18= 0 and skip to X19)**

1. How old were you when you first diagnosed with cholecystitis/cholelithiasis?

- 10 and less [X18a=1.2]
- 1 1-20 [X18a =1.0]
- 21-30 [X18a =0.8]
- 31-40 [X18a =0.6]
- 41-50 [X18a =0.4]
- 51 and older [X18a =0.2]

1. How many times in total have you experienced recurrence/relapse of cholecystitis/cholelithiasis?

- ____times(X18b=entered times)

1. How many days did it generally last for a recurrence/relapse of cholecystitis/cholelithiasis?

- ____days(X18c=entered days)

[X18bc=0.2, 0.4, 0.6, 0.8, 1.0, 1.2 respectively if X18b*X18c=1-180, 181-360, 361-720, 721-1080, 1081-1440, 1441 and over]

[X18=(X18a+X18bc)/2]

**X19: Chronic appendicitis/enteritis (if unchecked in rapid assessment, X19= 0 and skip to X20)**

1. How old were you when you first diagnosed with chronic appendicitis/enteritis?

- 10 and less [X19a=1.2]
- 1 1-20 [X19a =1.0]
- 21-30 [X19a =0.8]
- 31-40 [X19a =0.6]
- 41-50 [X19a =0.4]
- 51 and older [X19a =0.2]

1. How many times in total have you experienced recurrence/relapse of chronic appendicitis/enteritis?

- ____times(X19b=entered times)

1. How many days did it generally last for a recurrence/relapse of chronic appendicitis/enteritis?

- ____days(X19c=entered days)

[X19bc=0.2, 0.4, 0.6, 0.8, 1.0, 1.2 respectively if X19b * X19c =1-180, 181-360, 361-720, 721-1080, 1081-1440, 1441 and over]

[X19=(X19a+X19bc)/2]

**X20: Intestinal polyps (if unchecked in rapid assessment, X20= 0 and skip to X21)**

1. How old were you when you first diagnosed with intestinal polyps?

- 10 and less [X20a=1.2]
- 1 1-20 [X20 =1.0]
- 21-30 [X20 =0.8]
- 31-40 [X20 =0.6]
- 41-50 [X20 =0.4]
- 51 and older [X20 =0.2]

**X21: Pancreatitis (if unchecked in rapid assessment, X21= 0 and skip to X22)**

1. How old were you when you first diagnosed with pancreatitis?

- 10 and less [X21a=1.2]
- 1 1-20 [X21a =1.0]
- 21-30 [X21a =0.8]
- 31-40 [X21a =0.6]
- 41-50 [X21a =0.4]
- 51 and older [X21a =0.2]

1. How many times in total have you experienced recurrence/relapse of pancreatitis?

- ____times(X21b=entered times)

1. How many days did it generally last for a recurrence/relapse of pancreatitis?

- ____days(X21c=entered days)

[X21bc=0.2, 0.4, 0.6, 0.8, 1.0, 1.2 respectively if X21b*X21c=1-180, 181-360, 361-720, 721-1080, 1081-1440, 1441 and over]

[X21=(X21a+X21bc)/2]

**X22: Hemorrhoids (if unchecked in rapid assessment, X22= 0 and skip to X23)**

1. How old were you when you first diagnosed with hemorrhoids?

- 10 and less [X22a=1.2]
- 1 1-20 [X22a =1.0]
- 21-30 [X22a =0.8]
- 31-40 [X22a =0.6]
- 41-50 [X22a =0.4]
- 51 and older [X22a =0.2]

1. How many times in total have you experienced recurrence/relapse of hemorrhoids?

- ____times(X22b=entered times)

1. How many days did it generally last for a recurrence/relapse of hemorrhoids?

- ____days(X22c=entered days)

[X22bc=0.2, 0.4, 0.6, 0.8, 1.0, 1.2 respectively if X22b * X22c =1-180, 181-360, 361-720, 721-1080, 1081-1440, 1441 and over]

[X22=(X22a+X22bc)/2]

**X23: Chest distress/dyspnea (if unchecked in rapid assessment, X23= 0 and skip to X24)**

1. How old were you when you began to feel frequent chest distress/dyspnea?

- 10 and less [X23a=1.2]
- 1 1-20 [X23a =1.0]
- 21-30 [X23a =0.8]
- 31-40 [X23a =0.6]
- 41-50 [X23a =0.4]
- 51 and older [X23a =0.2]

1. How many times did you suffer from chest distress/dyspnea a year?

- 1-5[X23b=0.2]
- 6-10 [X23b =0.4]
- 11-15 [X23b =0.6]
- 16-20 [X23b =0.8]
- 21 -25[X23b =1.0]
- 26 and more[X23b =1.2]

[X23=(X23a+X23b)/2]

**X24: Chest pain (if unchecked in rapid assessment, X24= 0 and skip to X25)**

1. How old were you when you began to feel frequent chest pain?

- 10 and less [X24a=1.2]
- 1 1-20 [X24a =1.0]
- 21-30 [X24a =0.8]
- 31-40 [X24a =0.6]
- 41-50 [X24a =0.4]
- 51 and older [X24a =0.2]

1. How many times did you suffer from chest pain a year?

- 1-5[X24b=0.2]
- 6-10 [X24b =0.4]
- 11-15 [X24b =0.6]
- 16-20 [X24b =0.8]
- 21 -25[X24b =1.0]
- 26 and more[X24b =1.2]

[X24=(X24a+X24b)/2]

**X25: Cough (if unchecked in rapid assessment, X25= 0 and skip to X26)**

1. How old were you when you began to experience persistent cough?

- 10 and less [X25a=1.2]
- 1 1-20 [X25a =1.0]
- 21-30 [X25a =0.8]
- 31-40 [X25a =0.6]
- 41-50 [X25a =0.4]
- 51 and older [X25a =0.2]

1. How many times in total have you experienced recurrence/relapse of persistent cough?

- ____times(X25b=entered times)

1. How many days did it generally last for a recurrence/relapse of persistent cough?

- ____days(X25c=entered days)

[X25bc=0.2, 0.4, 0.6, 0.8, 1.0, 1.2 respectively if X25b*X25c=1-180, 181-360, 361-720, 721-1080, 1081-1440, 1441 and over]

[X25=(X25a+X25bc)/2]

**X26: Rhinobyon (if unchecked in rapid assessment, X26= 0 and skip to X27)**

1. How old were you when you began to experience frequent rhinobyon?

- 10 and less [X26a=1.2]
- 1 1-20 [X26a =1.0]
- 21-30 [X26a =0.8]
- 31-40 [X26a =0.6]
- 41-50 [X26a =0.4]
- 51 and older [X26a =0.2]

1. How many times in total have you experienced recurrence/relapse of rhinobyon?

- ____times(X26b=entered times)

1. How many days did it generally last for a recurrence/relapse of rhinobyon?

- ____days(X26c=entered days)

[X26bc=0.2, 0.4, 0.6, 0.8, 1.0, 1.2 respectively if X26b*X26c=1-180, 181-360, 361-720, 721-1080, 1081-1440, 1441 and over]

[X26=(X26a+X26bc)/2]

**X27: Running nose (if unchecked in rapid assessment, X27= 0 and skip to X28)**

1. How old were you when you began to experience persistent running nose?

- 10 and less [X27a=1.2]
- 1 1-20 [X27a =1.0]
- 21-30 [X27a =0.8]
- 31-40 [X27a =0.6]
- 41-50 [X27a =0.4]
- 51 and older [X27a =0.2]

1. How many times in total have you experienced recurrence/relapse of running nose?

- ____times(X27b=entered times)

1. How many days did it generally last for a recurrence/relapse of running nose?

- ____days(X27c=entered days)

[X27bc=0.2, 0.4, 0.6, 0.8, 1.0, 1.2 respectively if X27b*X27c=1-180, 181-360, 361-720, 721-1080, 1081-1440, 1441 and over]

[X27=(X27a+X27bc)/2]

**X28: Tuberculosis (if unchecked in rapid assessment, X28= 0 and skip to X29)**

1. How old were you when you first diagnosed with tuberculosis?

- 10 and less [X28a=1.2]
- 1 1-20 [X28a =1.0]
- 21-30 [X28a =0.8]
- 31-40 [X28a =0.6]
- 41-50 [X28a =0.4]
- 51 and older [X28a =0.2]

1. How many times in total have you experienced recurrence/relapse of tuberculosis?

- ____times(X28b=entered times)

1. How many days did it generally last for a recurrence/relapse of tuberculosis?

- ____days(X28c=entered days)

[X28bc=0.2, 0.4, 0.6, 0.8, 1.0, 1.2 respectively if X28b*X28c=1-180, 181-360, 361-720, 721-1080, 1081-1440, 1441 and over]

[X28=(X28a+X28bc)/2]

**X29: Chronic obstructive pulmonary disease (if unchecked in rapid assessment, X29= 0 and skip to X30)**

1. How old were you when you first diagnosed with chronic obstructive pulmonary disease?

- 10 and less [X29a=1.2]
- 1 1-20 [X29a =1.0]
- 21-30 [X29a =0.8]
- 31-40 [X29a =0.6]
- 41-50 [X29a =0.4]
- 51 and older [X29a =0.2]

1. How many times in total have you experienced recurrence/relapse of chronic obstructive pulmonary disease?

- ____times(X29b=entered times)

1. How many days did it generally last for a recurrence/relapse of chronic obstructive pulmonary disease?

- ____days(X29c=entered days)

[X29bc=0.2, 0.4, 0.6, 0.8, 1.0, 1.2 respectively if X29b * X29c =1-180, 181-360, 361-720, 721-1080, 1081-1440, 1441 and over]

[X29=(X29a+ X29bc)/2]

**X30: Asthma** **(if unchecked in rapid assessment, X30= 0 and skip to X31)**

1. How old were you when you first diagnosed with asthma?

- 10 and less [X30a=1.2]
- 1 1-20 [X30a =1.0]
- 21-30 [X30a =0.8]
- 31-40 [X30a =0.6]
- 41-50 [X30a =0.4]
- 51 and older [X30a =0.2]

1. How many times in total have you experienced recurrence/relapse of asthma?

- ____times(X30b=entered times)

1. How many days did it generally last for a recurrence/relapse of asthma?

- ____days(X30c=entered days)

[X30bc=0.2, 0.4, 0.6, 0.8, 1.0, 1.2 respectively if X30b * X30c =1-180, 181-360, 361-720, 721-1080, 1081-1440, 1441 and over]

[X30=( X30a + X30bc)/2]

**X31: Pneumonia** **(if unchecked in rapid assessment, X31= 0 and skip to X32)**

1. How old were you when you first diagnosed with pneumonia?

- 10 and less [X31a=1.2]
- 1 1-20 [X31a =1.0]
- 21-30 [X31a =0.8]
- 31-40 [X31a =0.6]
- 41-50 [X31a =0.4]
- 51 and older [X31a =0.2]

1. How many times in total have you experienced recurrence/relapse of pneumonia?

- ____times(X31b=entered times)

1. How many days did it generally last for a recurrence/relapse of pneumonia?

- ____days(X31c=entered days)

[X31bc=0.2, 0.4, 0.6, 0.8, 1.0, 1.2 respectively if X31b * X31c =1-180, 181-360, 361-720, 721-1080, 1081-1440, 1441 and over]

[X31=( X31a + X31bc)/2]

**X32: Bronchitis** **(if unchecked in rapid assessment, X32= 0 and skip to X33)**

1. How old were you when you first diagnosed with bronchitis?

- 10 and less [X32a=1.2]
- 1 1-20 [X32a =1.0]
- 21-30 [X32a =0.8]
- 31-40 [X32a =0.6]
- 41-50 [X32a =0.4]
- 51 and older [X32a =0.2]

1. How many times in total have you experienced recurrence/relapse of bronchitis?

- ____times(X32b=entered times)

1. How many days did it generally last for a recurrence/relapse of bronchitis?

- ____days(X32c=entered days)

[X32bc=0.2, 0.4, 0.6, 0.8, 1.0, 1.2 respectively if X32b * X32c =1-180, 181-360, 361-720, 721-1080, 1081-1440, 1441 and over]

[X32=( X32a + X32bc)/2]

**X33: Emphysema** **(if unchecked in rapid assessment, X33= 0 and skip to X34)**

1. How old were you when you first diagnosed with bronchitis?

- 10 and less [X33a=1.2]
- 1 1-20 [X33a =1.0]
- 21-30 [X33a =0.8]
- 31-40 [X33a =0.6]
- 41-50 [X33a =0.4]
- 51 and older [X33a =0.2]

1. How many times in total have you experienced recurrence/relapse of bronchitis?

- ____times(X33b=entered times)

1. How many days did it generally last for a recurrence/relapse of bronchitis?

- ____days(X33c =entered days)

[X33bc=0.2, 0.4, 0.6, 0.8, 1.0, 1.2 respectively if X33b * X33c =1-180, 181-360, 361-720, 721-1080, 1081-1440, 1441 and over]

[X33=( X33a + X33bc)/2]

**X34: Silicosis** **(if unchecked in rapid assessment, X34= 0 and skip to X35)**

1. How old were you when you first diagnosed with bronchitis?

- 10 and less [X34a=1.2]
- 1 1-20 [X34a =1.0]
- 21-30 [X34a =0.8]
- 31-40 [X34a =0.6]
- 41-50 [X34a =0.4]
- 51 and older [X34a =0.2]

1. How many times in total have you experienced recurrence/relapse of bronchitis?

- ____times(X34b=entered times)

1. How many days did it generally last for a recurrence/relapse of bronchitis?

- ____days(X34c =entered days)

[X34bc=0.2, 0.4, 0.6, 0.8, 1.0, 1.2 respectively if X34b * X34c =1-180, 181-360, 361-720, 721-1080, 1081-1440, 1441 and over]

[X34=( X34a + X34bc)/2]

**X35: Pneumoconiosis (if unchecked in rapid assessment, X35= 0 and skip to X36)**

1. How old were you when you first diagnosed with pneumoconiosis?

- 10 and less [X35a=1.2]
- 1 1-20 [X35a =1.0]
- 21-30 [X35a =0.8]
- 31-40 [X35a =0.6]
- 41-50 [X35a =0.4]
- 51 and older [X35a =0.2]

1. How many times in total have you experienced recurrence/relapse of pneumoconiosis?

- ____times(X35b=entered times)

1. How many days did it generally last for a recurrence/relapse of pneumoconiosis?

- ____days(X35c =entered days)

[X35bc=0.2, 0.4, 0.6, 0.8, 1.0, 1.2 respectively if X35b * X35c =1-180, 181-360, 361-720, 721-1080, 1081-1440, 1441 and over]

[X35=( X35a + X35bc)/2]

**X36: Chronic rhinitis (if unchecked in rapid assessment, X36= 0 and skip to X37)**

1. How old were you when you first diagnosed with chronic rhinitis?

- 10 and less [X36a=1.2]
- 1 1-20 [X36a =1.0]
- 21-30 [X36a =0.8]
- 31-40 [X36a =0.6]
- 41-50 [X36a =0.4]
- 51 and older [X36a =0.2]

1. How many times in total have you experienced recurrence/relapse of chronic rhinitis?

- ____times(X36b=entered times)

1. How many days did it generally last for a recurrence/relapse of chronic rhinitis?

- ____days(X36c =entered days)

[X36bc=0.2, 0.4, 0.6, 0.8, 1.0, 1.2 respectively if X36b * X36c =1-180, 181-360, 361-720, 721-1080, 1081-1440, 1441 and over]

[X36=( X36a + X36bc)/2]

**X37: Epstein-Barr virus infection (if unchecked in rapid assessment, X37= 0 and skip to X38)**

1. How old were you when you first diagnosed with Epstein-Barr virus infection?

- 10 and less [X37a=1.2]
- 1 1-20 [X37a =1.0]
- 21-30 [X37a =0.8]
- 31-40 [X37a =0.6]
- 41-50 [X37a =0.4]
- 51 and older [X37a =0.2]

1. How many times in total have you experienced recurrence/relapse of Epstein-Barr virus infection?

- ____times(X37b=entered times)

1. How many days did it generally last for a recurrence/relapse of Epstein-Barr virus infection?

- ____days(X37c =entered days)

[X37bc=0.2, 0.4, 0.6, 0.8, 1.0, 1.2 respectively if X37b * X37c =1-180, 181-360, 361-720, 721-1080, 1081-1440, 1441 and over]

[X37= (X37a + X37bc)/2]

**X38: Breast pain (if unchecked in rapid assessment, X38= 0 and skip to X39)**

1. How old were you when you began to feel frequent breast pain?

- 10 and less [X38a=1.2]
- 1 1-20 [X38a =1.0]
- 21-30 [X38a =0.8]
- 31-40 [X38a =0.6]
- 41-50 [X38a =0.4]
- 51 and older [X38a =0.2]

1. How many times in total have you experienced recurrence/relapse of breast pain?

- ____times(X38b=entered times)

1. How many days did it generally last for a recurrence/relapse of breast pain?

- ____days(X38c =entered days)

[X38bc=0.2, 0.4, 0.6, 0.8, 1.0, 1.2 respectively if X38b * X38c =1-180, 181-360, 361-720, 721-1080, 1081-1440, 1441 and over]

[X38= (X38a + X38bc)/2]

**X39: Breast mass (if unchecked in rapid assessment, X39= 0 and skip to X40)**

1. How old were you when you began to feel breast mass?

- 10 and less [X39a=1.2]
- 1 1-20 [X39a =1.0]
- 21-30 [X39a =0.8]
- 31-40 [X39a =0.6]
- 41-50 [X39a =0.4]
- 51 and older [X39a =0.2]

1. How many times in total have you experienced recurrence/relapse of breast mass?

- ____times(X39b=entered times)

1. How many days did it generally last for a recurrence/relapse of breast mass?

- ____days(X39c =entered days)

[X39bc=0.2, 0.4, 0.6, 0.8, 1.0, 1.2 respectively if X39b * X39c =1-180, 181-360, 361-720, 721-1080, 1081-1440, 1441 and over]

[X39= (X39a + X39bc)/2]

**X40: Nipple discharge (if unchecked in rapid assessment, X40= 0 and skip to X41)**

1. How old were you when you began to present nipple discharge?

- 10 and less [X40a=1.2]
- 1 1-20 [X40a =1.0]
- 21-30 [X40a =0.8]
- 31-40 [X40a =0.6]
- 41-50 [X40a =0.4]
- 51 and older [X40a =0.2]

1. How many times in total have you experienced recurrence/relapse of nipple discharge?

- ____times(X40b=entered times)

1. How many days did it generally last for a recurrence/relapse of nipple discharge?

- ____days(X40c =entered days)

[X40bc=0.2, 0.4, 0.6, 0.8, 1.0, 1.2 respectively if X40b * X40c =1-180, 181-360, 361-720, 721-1080, 1081-1440, 1441 and over]

[X40= (X40a + X40bc)/2]

**X41: Colporrhagia (if unchecked in rapid assessment, X41= 0 and skip to X42)**

1. How old were you when you began to present abnormal colporrhagia?

- 10 and less [X41a=1.2]
- 1 1-20 [X41a =1.0]
- 21-30 [X41a =0.8]
- 31-40 [X41a =0.6]
- 41-50 [X41a =0.4]
- 51 and older [X41a =0.2]

1. How many times in total have you experienced recurrence/relapse of abnormal colporrhagia?

- ____times(X41b=entered times)

1. How many days did it generally last for a recurrence/relapse of abnormal colporrhagia?

- ____days(X41c =entered days)

[X41bc=0.2, 0.4, 0.6, 0.8, 1.0, 1.2 respectively if X41b * X41c =1-180, 181-360, 361-720, 721-1080, 1081-1440, 1441 and over]

[X41= (X41a + X41b+ X41c)/3]

**X42: Abnormal leucorrhea (if unchecked in rapid assessment, X42= 0 and skip to X43)**

1. How old were you when you began to see abnormal leucorrhea?

- 10 and less [X42a=1.2]
- 1 1-20 [X42a =1.0]
- 21-30 [X42a =0.8]
- 31-40 [X42a =0.6]
- 41-50 [X42a =0.4]
- 51 and older [X42a =0.2]

1. How many times in total have you experienced recurrence/relapse of abnormal leucorrhea?

- ____times(X42b=entered times)

1. How many days did it generally last for a recurrence/relapse of abnormal leucorrhea?

- ____days(X42c =entered days)

[X42bc=0.2, 0.4, 0.6, 0.8, 1.0, 1.2 respectively if X42b * X42c =1-180, 181-360, 361-720, 721-1080, 1081-1440, 1441 and over]

[X42= (X42a + X42b+ X42c)/3]

**X43: Pelvic mass (if unchecked in rapid assessment, X43= 0 and skip to X44)**

1. How old were you when you began to feel pelvic mass?

- 10 and less [X43a=1.2]
- 1 1-20 [X43a =1.0]
- 21-30 [X43a =0.8]
- 31-40 [X43a =0.6]
- 41-50 [X43a =0.4]
- 51 and older [X43a =0.2]

1. How many times in total have you experienced recurrence/relapse of pelvic mass?

- ____times(X43b=entered times)

1. How many days did it generally last for a recurrence/relapse of pelvic mass?

- ____days(X43c =entered days)

[X43bc=0.2, 0.4, 0.6, 0.8, 1.0, 1.2 respectively if X43b * X43c =1-180, 181-360, 361-720, 721-1080, 1081-1440, 1441 and over]

[X43=( X43a + X43b +X43bc)/3]

**X44: Hypogastralgia (if unchecked in rapid assessment, X44= 0 and skip to X45)**

1. How old were you when you began to feel hypogastralgia?

- 10 and less [X44a=1.2]
- 1 1-20 [X44a =1.0]
- 21-30 [X44a =0.8]
- 31-40 [X44a =0.6]
- 41-50 [X44a =0.4]
- 51 and older [X44a =0.2]

1. How many times in total have you experienced recurrence/relapse of hypogastralgia?

- ____times(X44b=entered times)

1. How many days did it generally last for a recurrence/relapse of hypogastralgia?

- ____days(X44c =entered days)

[X44bc=0.2, 0.4, 0.6, 0.8, 1.0, 1.2 respectively if X44b * X44c =1-180, 181-360, 361-720, 721-1080, 1081-1440, 1441 and over]

[X43=( X43a + X44b+ X44bc)/3]

**X45: Lumbago pain and tenesmus (if unchecked in rapid assessment, X45= 0 and skip to X46)**

1. How old were you when you began to feel lumbago pain and tenesmus?

- 10 and less [X45a=1.2]
- 1 1-20 [X45a =1.0]
- 21-30 [X45a =0.8]
- 31-40 [X45a =0.6]
- 41-50 [X45a =0.4]
- 51 and older [X45a =0.2]

1. How many times in total have you experienced recurrence/relapse of lumbago pain and tenesmus?

- ____times(X45b=entered times)

1. How many days did it generally last for a recurrence/relapse of lumbago pain and tenesmus?

- ____days(X45c =entered days)

[X45bc=0.2, 0.4, 0.6, 0.8, 1.0, 1.2 respectively if X45b * X45c =1-180, 181-360, 361-720, 721-1080, 1081-1440, 1441 and over]

[X45=( X45a + X45b+ X45bc)/3]

**X46: Mastitis (if unchecked in rapid assessment, X46= 0 and skip to X47)**

1. How old were you when you first diagnosed with mastitis?

- 10 and less [X46a=1.2]
- 1 1-20 [X46a =1.0]
- 21-30 [X46a =0.8]
- 31-40 [X46a =0.6]
- 41-50 [X46a =0.4]
- 51 and older [X46a =0.2]

1. How many times in total have you experienced recurrence/relapse of mastitis?

- ____times(X46b=entered times)

1. How many days did it generally last for a recurrence/relapse of mastitis?

- ____days(X46c =entered days)

[X46bc=0.2, 0.4, 0.6, 0.8, 1.0, 1.2 respectively if X46b * X46c =1-180, 181-360, 361-720, 721-1080, 1081-1440, 1441 and over]

[X46=( X46a + X46bc)/2]

**X47: Hyperplasia of mammary glands (if unchecked in rapid assessment, X47= 0 and skip to X48)**

1. How old were you when you first diagnosed with hyperplasia of mammary glands?

- 10 and less [X47a=1.2]
- 1 1-20 [X47a =1.0]
- 21-30 [X47a =0.8]
- 31-40 [X47a =0.6]
- 41-50 [X47a =0.4]
- 51 and older [X47a =0.2]

1. How many times in total have you experienced recurrence/relapse of hyperplasia of mammary glands?

- ____times(X47b=entered times)

1. How many days did it generally last for a recurrence/relapse of hyperplasia of mammary glands?

- ____days(X47c =entered days)

[X47bc=0.2, 0.4, 0.6, 0.8, 1.0, 1.2 respectively if X47b * X47c =1-180, 181-360, 361-720, 721-1080, 1081-1440, 1441 and over]

[X47=( X47a + X47bc)/2]

**X48: Fibroadenoma of breast (if unchecked in rapid assessment, X48= 0 and skip to X49)**

1. How old were you when you first diagnosed with fibroadenoma of breast?

- 10 and less [X48a=1.2]
- 1 1-20 [X48a =1.0]
- 21-30 [X48a =0.8]
- 31-40 [X48a =0.6]
- 41-50 [X48a =0.4]
- 51 and older [X48a =0.2]

1. How many times in total have you experienced recurrence/relapse of fibroadenoma of breast?

- ____times(X48b=entered times)

1. How many days did it generally last for a recurrence/relapse of fibroadenoma of breast?

- ____days(X48c =entered days)

[X48bc=0.2, 0.4, 0.6, 0.8, 1.0, 1.2 respectively if X48b * X48c =1-180, 181-360, 361-720, 721-1080, 1081-1440, 1441 and over]

[X48=( X48a + X48bc)/2]

**X49: Galactoma (if unchecked in rapid assessment, X49= 0 and skip to X50)**

1. How old were you when you first diagnosed with galactoma?

- 10 and less [X49a=1.2]
- 1 1-20 [X49a =1.0]
- 21-30 [X49a =0.8]
- 31-40 [X49a =0.6]
- 41-50 [X49a =0.4]
- 51 and older [X49a =0.2]

1. How many times in total have you experienced recurrence/relapse of galactoma?

- ____times(X49b=entered times)

1. How many days did it generally last for a recurrence/relapse of galactoma?

- ____days(X49c=entered days)

[X49bc=0.2, 0.4, 0.6, 0.8, 1.0, 1.2 respectively if X49b * X49c =1-180, 181-360, 361-720, 721-1080, 1081-1440, 1441 and over]

[X49=( X51a + X49bc)/2]

**X50: Gynecological inflammation（cervicitis/ vaginitis/ pelvic inflammatory/ endometritis）(if unchecked in rapid assessment, X50= 0 and skip to X51)**

1. How old were you when you first diagnosed with gynecological inflammation?

- 10 and less [X50a=1.2]
- 1 1-20 [X50a =1.0]
- 21-30 [X50a =0.8]
- 31-40 [X50a =0.6]
- 41-50 [X50a =0.4]
- 51 and older [X50a =0.2]

1. How many times in total have you experienced recurrence/relapse of gynecological inflammation?

- ____times(X50b=entered times)

1. How many days did it generally last for a recurrence/relapse of gynecological inflammation?

- ____days(X50c=entered days)

[X50bc=0.2, 0.4, 0.6, 0.8, 1.0, 1.2 respectively if X50b * X50c =1-180, 181-360, 361-720, 721-1080, 1081-1440, 1441 and over]

[X50=( X50a + X50bc)/2]

**X51: STIs (condyloma acuminatum/ syphilis/ genital herpes/ gonorrhea/HIV) (if unchecked in rapid assessment, X51= 0 and skip to X52)**

1. How old were you when you first diagnosed with STIs?

- 10 and less [X51a=1.2]
- 1 1-20 [X51a =1.0]
- 21-30 [X51a =0.8]
- 31-40 [X51a =0.6]
- 41-50 [X51a =0.4]
- 51 and older [X51a =0.2]

1. How many times in total have you experienced recurrence/relapse of STIs?

- ____times(X51b=entered times)

1. How many days did it generally last for a recurrence/relapse of STIs?

- ____days(X51c=entered days)

[X51bc=0.2, 0.4, 0.6, 0.8, 1.0, 1.2 respectively if X51b * X51c =1-180, 181-360, 361-720, 721-1080, 1081-1440, 1441 and over]

[X51=( X51a + X51bc)/2]

**X52: HPV (human papillomavirus) infection (if unchecked in rapid assessment, X52= 0 and skip to X53)**

1. How old were you when you first diagnosed with HPV?

- 10 and less [X52a=1.2]
- 1 1-20 [X52a =1.0]
- 21-30 [X52a =0.8]
- 31-40 [X52a =0.6]
- 41-50 [X52a =0.4]
- 51 and older [X52a =0.2]

1. How many times in total have you experienced recurrence/relapse of HPV infection?

- ____times(X52b=entered times)

1. How many days did it generally last for a recurrence/relapse of HPV infection?

- ____days(X52c=entered days)

[X52bc=0.2, 0.4, 0.6, 0.8, 1.0, 1.2 respectively if X52b * X52c =1-180, 181-360, 361-720, 721-1080, 1081-1440, 1441 and over]

[X52=( X52a + X52bc)/2]

**X53: Irregular menstruation/dysmenorrhea (if unchecked in rapid assessment, X53= 0 and skip to X55)**

1. How old were you when you first diagnosed with irregular menstruation/ dysmenorrhea?

- 10 and less [X53a=1.2]
- 1 1-20 [X53a =1.0]
- 21-30 [X53a =0.8]
- 31-40 [X53a =0.6]
- 41-50 [X53a =0.4]
- 51 and older [X53a =0.2]

1. How many times in total have you experienced recurrence/relapse of irregular menstruation/dysmenorrhea?

- ____times(X53b=entered times)

1. How many days did it generally last for a recurrence/relapse of irregular menstruation/ dysmenorrhea?

- ____days(X53c=entered days)

[X53bc=0.2, 0.4, 0.6, 0.8, 1.0, 1.2 respectively if X53b * X53c =1-180, 181-360, 361-720, 721-1080, 1081-1440, 1441 and over]

[X53=( X53a + X53bc)/2]

**X55: Uterine fibroid (if unchecked in rapid assessment, X55= 0 and skip to X56)**

1. How old were you when you first diagnosed with uterine fibroid?

- 10 and less [X55=1.2]
- 1 1-20 [X55=1.0]
- 21-30 [X55=0.8]
- 31-40 [X55=0.6]
- 41-50 [X55=0.4]
- 51 and older [X55=0.2]

**X56: Cervical cyst (if unchecked in rapid assessment, X56= 0 and skip to X57)**

1. How old were you when you first diagnosed with cervical cyst?

- 10 and less [X56=1.2]
- 1 1-20 [X56=1.0]
- 21-30 [X56=0.8]
- 31-40 [X56=0.6]
- 41-50 [X56=0.4]
- 51 and older [X56=0.2]

**X57: Ovarian cyst (if unchecked in rapid assessment, X57= 0 and skip to X59)**

1. How old were you when you first diagnosed with ovarian cyst?

- 10 and less [X57=1.2]
- 1 1-20 [X57=1.0]
- 21-30 [X57=0.8]
- 31-40 [X57=0.6]
- 41-50 [X57=0.4]
- 51 and older [X57=0.2]

**X59:** **Diabetes** **(if unchecked in rapid assessment, X59= 0 and skip to X60)**

1. How old were you when you first diagnosed with diabetes?

- 10 and less [X59=1.2]
- 1 1-20 [X59=1.0]
- 21-30 [X59=0.8]
- 31-40 [X59=0.6]
- 41-50 [X59=0.4]
- 51 and older [X59=0.2]

**X60:** **Hypertension** **(if unchecked in rapid assessment, X60= 0 and skip to X61)**

1. How old were you when you first diagnosed with hypertension?

- 10 and less [X60=1.2]
- 1 1-20 [X60=1.0]
- 21-30 [X60=0.8]
- 31-40 [X60=0.6]
- 41-50 [X60=0.4]
- 51 and older [X60=0.2]

**X61:** **Hyperlipidemia** **(if unchecked in rapid assessment, X61= 0 and skip to X62)**

1. How old were you when you first diagnosed with hyperlipidemia?

- 10 and less [X61=1.2]
- 1 1-20 [X61=1.0]
- 21-30 [X61=0.8]
- 31-40 [X61=0.6]
- 41-50 [X61=0.4]
- 51 and older [X61=0.2]

**X62:** **Rheumatoid** **arthritis (if unchecked in rapid assessment, X62= 0 and skip to X63)**

1. How old were you when you first diagnosed with rheumatoid arthritis?

- 10 and less [X62a=1.2]
- 1 1-20 [X62a =1.0]
- 21-30 [X62a =0.8]
- 31-40 [X62a =0.6]
- 41-50 [X62a =0.4]
- 51 and older [X62a =0.2]

1. How many times in total have you experienced recurrence/relapse of rheumatoid arthritis?

- ____times(X62b=entered times)

1. How many days did it generally last for a recurrence/relapse of rheumatoid arthritis?

- ____days(X62c=entered days)

[X62bc=0.2, 0.4, 0.6, 0.8, 1.0, 1.2 respectively if X62b * X62c =1-180, 181-360, 361-720, 721-1080, 1081-1440, 1441 and over]

[X62=( X62a + X62bc)/2]

**X63: Systemic lupus erythematosus (if unchecked in rapid assessment, X63= 0 and skip to X64)**

1. How old were you when you first diagnosed with systemic lupus erythematosus?

- 10 and less [X63a=1.2]
- 1 1-20 [X63a =1.0]
- 21-30 [X63a =0.8]
- 31-40 [X63a =0.6]
- 41-50 [X63a =0.4]
- 51 and older [X63a =0.2]

1. How many times in total have you experienced recurrence/relapse of systemic lupus erythematosus?

- ____times(X63b=entered times)

1. How many days did it generally last for a recurrence/relapse of systemic lupus erythematosus?

- ____days(X63c=entered days)

[X63bc=0.2, 0.4, 0.6, 0.8, 1.0, 1.2 respectively if X63b * X63c =1-180, 181-360, 361-720, 721-1080, 1081-1440, 1441 and over]

[X63=( X63a + X63bc)/2]

**X64: Insomnia/ dreaminess (if unchecked in rapid assessment, X64= 0 and skip to X66)**

1. How old were you when you first experienced frequent insomnia/ dreaminess?

- 10 and less [X64a=1.2]
- 1 1-20 [X64a =1.0]
- 21-30 [X64a =0.8]
- 31-40 [X64a =0.6]
- 41-50 [X64a =0.4]
- 51 and older [X64a =0.2]

1. How many times in total have you experienced recurrence/relapse of insomnia/ dreaminess?

- ____times(X64b=entered times)

1. How many days did it generally last for a recurrence/relapse of insomnia/ dreaminess?

- ____days(X64c=entered days)

[X64bc=0.2, 0.4, 0.6, 0.8, 1.0, 1.2 respectively if X64b * X64c =1-180, 181-360, 361-720, 721-1080, 1081-1440, 1441 and over]

[X64=( X64a + X64bc)/2]

**X66: Overweight/obesity (if unchecked in rapid assessment, X66= 0 and skip to X67)**

1. How old were you when you became overweight/obesity?

- 10 and less [X66=1.2]
- 1 1-20 [X66=1.0]
- 21-30 [X66=0.8]
- 31-40 [X66=0.6]
- 41-50 [X66=0.4]
- 51 and older [X66=0.2]

**X67: Underweight (if unchecked in rapid assessment, X67= 0 and skip to X68)**

1. How old were you when you became underweight?

- 10 and less [X67=1.2]
- 1 1-20 [X67=1.0]
- 21-30 [X67=0.8]
- 31-40 [X67=0.6]
- 41-50 [X67=0.4]
- 51 and older [X67=0.2]

**X68: Laxatives (if unchecked in rapid assessment, X68= 0 and skip to X69)**

1. How many times in total have you used laxatives?

- 10 and less [X68=0.2]
- 1 1-20 [X68=0.4]
- 21-30 [X68=0.6]
- 31-40 [X68=0.8]
- 41-50 [X68=1.0]
- 51 and more [X68=1.2]

**X69: Vitamin B12 (if unchecked in rapid assessment, X69= 0 and skip to X70)**

1. How many days in total have you used Vitamin B12?

- 10 and less [X69=0.2]
- 1 1-20 [X69=0.4]
- 21-30 [X69=0.6]
- 31-40 [X69=0.8]
- 41-50 [X69=1.0]
- 51 and more [X69=1.2]

**X70: Cholecystectomy (if unchecked in rapid assessment, X70= 0 and skip to X71)**

1. How old were you when you received cholecystectomy?

- 10 and less [X70=1.2]
- 1 1-20 [X70=1.0]
- 21-30 [X70=0.8]
- 31-40 [X70=0.6]
- 41-50 [X70=0.4]
- 51 and older [X70=0.2]

**X71: Gastrectomy (if unchecked in rapid assessment, X71= 0 and skip to X72)**

1. How old were you when you received gastrectomy?

- 10 and less [X71=1.2]
- 1 1-20 [X71=1.0]
- 21-30 [X71=0.8]
- 31-40 [X71=0.6]
- 41-50 [X71=0.4]
- 51 and older [X71=0.2]

**X72: Calcium channel blocker (Nifedipine/Verapamil/Diltiazem) (if unchecked in rapid assessment, X72= 0 and skip to X73)**

1. How many years have you used calcium channel blocker?

- Less than 1 [X72=0.2]
- 1-2 [X72=0.4]
- 3-4 [X72=0.6]
- 5-6 [X72=0.8]
- 7-8 [X72=1.0]
- 9 and more [X72=1.2]

**X73: Estrogen (if unchecked in rapid assessment, X73= 0 and skip to X92)**

1. How many months have you used estrogen?

- 1-3 [X73=0.2]
- 4-6 [X73=0.4]
- 7-9 [X73=0.6]
- 10-12 [X73=0.8]
- 13-15 [X73=1.0]
- 16 and more [X73=1.2]

**X92: Pickled food (if unchecked in rapid assessment, X92= 0 and skip to X93)**

1. Which of the following types of pickled foods do you eat frequently (please make multiple checks if applicable)?

- Bacon/chicken/duck/goose/fish [X92a1=0.3 if checked, else 0]
- Pickles/stew chili/bean sauce [X92a2=0.3 if checked, else 0]
- Pickled eggs/pickled duck [X92a3 =0.3 if checked, else 0]
- others[X92a4 =0.3 if checked, else 0]

1. Which season(s) of the year do you eat pickled foods frequently (please make multiple checks if applicable)?

- Spring [X92b1=0.3 if checked, else 0]
- Summer[X92b2 =0.3 if checked, else 0]
- Autumn[X92b3 =0.3 if checked, else 0]
- Winter[X92b4 =0.3 if checked, else 0]

1. How many times do you eat pickled foods a month on average?

- 1-6 [X92c =0.2]
- 7-12 [X92c =0.4]
- 13-18[X92c =0.6]
- 19-24 [X92c =0.8]
- 25-30 [X92c =1.0]
- 31 and more [X92c =1.2]

X92a= X92a1+ X92a2+ X92a3+ X92a4, X92b= X92b1+ X92b2+ X92b3+ X92b4

[X92=( X92a + X92b+X92c)/3]

**X93: Dried** **food (if unchecked in rapid assessment, X93= 0 and skip to X94)**

1. Which of the following types of dried foods do you eat frequently (please make multiple checks if applicable)?

- Fish[X93a1=0.24 if checked, else 0]
- meat[X93a2=0.24 if checked, else 0]
- vegetables[X93a3 =0.24 if checked, else 0]
- fruits[X93a4 =0.24 if checked, else 0]
- others[X93a5 =0.24 if checked, else 0]

1. Which season(s) of the year do you eat dried foods frequently (please make multiple checks if applicable)?

- Spring [X93b1=0.3 if checked, else 0]
- Summer[X93b2=0.3 if checked, else 0]
- Autumn[X93b3 =0.3 if checked, else 0]
- Winter[X93b4 =0.3 if checked, else 0]

1. How many times do you eat dried foods a month on average?

- 1-6 [X93c =0.2]
- 7-12 [X93c =0.4]
- 13-18[X93c =0.6]
- 19-24 [X93c =0.8]
- 25-30 [X93c =1.0]
- 31 and more [X93c =1.2]

[X93a= X93a1+ X93a2+ X93a3+ X93a4, X93b= X93b1+ X93b2+ X93b3+ X93b4]

[X93=( X93a + X93b+ X93c)/3]

**X94: Smoked** **food (if unchecked in rapid assessment, X94= 0 and skip to X95)**

1. Which season(s) of the year do you eat smoked foods frequently (please make multiple checks if applicable)?

- Spring [X94a1=0.3 if checked, else 0]
- Summer[X94a2=0.3 if checked, else 0]
- Autumn[X94a3 =0.3 if checked, else 0]
- Winter[X94a4 =0.3 if checked, else 0]

1. How many times do you eat smoked foods a month on average?

- 1-6 [X94b =0.2]
- 7-12 [X94b =0.4]
- 13-18[X94b =0.6]
- 19-24 [X94b =0.8]
- 25-30 [X94b =1.0]
- 31 and more [X94b =1.2]

[X94a= X94a1+ X94a2+ X94a3+ X94a4]

[X94=( X94a + X94b)/2]

**X95: Fried food (if unchecked in rapid assessment, X95= 0 and skip to X96)**

1. Which of the following types of fried foods do you eat frequently (please make multiple checks if applicable)?

- Fried fish/meat[X95a1=0.24 if checked, else 0]
- Fried vegetables [X95a2=0.24 if checked, else 0]
- Fried fruits [X95a3 =0.24 if checked, else 0]
- Fried pasta [X95a4 =0.24 if checked, else 0]
- others[X95a5 =0.24 if checked, else 0]

1. What season of the year do you eat fried foods frequently(please make multiple checks if applicable)?

- Spring [X95b1=0.3 if checked, else 0]
- Summer[X95b2=0.3 if checked, else 0]
- Autumn[X95b3 =0.3 if checked, else 0]
- Winter[X95b4 =0.3 if checked, else 0]

1. How many times do you eat fried foods a month on average?

- 1-6 [X95c =0.2]
- 7-12 [X95c =0.4]
- 13-18[X95c =0.6]
- 19-24 [X95c =0.8]
- 25-30 [X95c =1.0]
- 31 and more [X95c =1.2]

[X95a= X95a1+ X95a2+ X95a3+ X95a4, X95b= X95b1+ X95b2+ X95b3+ X95b4]

[X95=( X95a + X95b + X95c)/3]

**X96: Spicy** **food (if unchecked in rapid assessment, X96= 0 and skip to X97)**

1. Which of the following types of spicy foods do you eat most frequently?

- Mild spicy food[X96a=0.3]
- Moderate spicy food [X96a=0.3]
- Highly spicy food [X96a=0.3]
- Very spicy food[X96a=0.3]

1. What season of the year do you eat spicy foods frequently(please make multiple checks if applicable)?

- Spring [X96b1=0.3 if checked, else 0]
- Summer[X96b2=0.3 if checked, else 0]
- Autumn[X96b3=0.3 if checked, else 0]
- Winter[X96b4=0.3 if checked, else 0]

1. How many times do you eat spicy foods a month on average?

- 1-6 [X96c =0.2]
- 7-12 [X96c =0.4]
- 13-18[X96c =0.6]
- 19-24 [X96c =0.8]
- 25-30 [X96c =1.0]
- 31 and more [X96c =1.2]

[X96b= X96b1+ X96b2+ X96b3+ X96b4]

[X96=( X96a + X96b+ X96c)/3]

**X97: Processed** **food (if unchecked in rapid assessment, X97= 0 and skip to X98)**

1. What season of the year do you eat processed foods frequently(please make multiple checks if applicable)?

- Spring [X97a1=0.3 if checked, else 0]
- Summer[X97a2=0.3 if checked, else 0]
- Autumn[X97a3=0.3 if checked, else 0]
- Winter[X97a4=0.3 if checked, else 0]

1. How many times do you eat processed foods a month on average?

- 1-6 [X97b =0.2]
- 7-12 [X97b =0.4]
- 13-18[X97b =0.6]
- 19-24 [X97b =0.8]
- 25-30 [X97b =1.0]
- 31 and more [X97b =1.2]

[X97a= X97a1+ X97a2+ X97a3+ X97a4]

[X97= ( X97a + X97b)/3]

**X98: Hard** **food (if unchecked in rapid assessment, X98= 0 and skip to X99)**

1. How many times do you eat hard foods a month on average?

- 1-6 [X98 =0.2]
- 7-12 [X98 =0.4]
- 13-18[X98 =0.6]
- 19-24 [X98 =0.8]
- 25-30 [X98 =1.0]
- 31 and more [X98 =1.2]

**X99:** **Fermented** **food (if unchecked in rapid assessment, X99= 0 and skip to X100)**

1. How many times do you eat fermented foods a month on average?

- 1-6 [X99 =0.2]
- 7-12 [X99 =0.4]
- 13-18[X99=0.6]
- 19-24 [X99 =0.8]
- 25-30 [X99 =1.0]
- 31 and more [X99 =1.2]

**X100:** **Leftovers (if unchecked in rapid assessment, X100= 0 and skip to X101)**

1. Which of the following types of leftovers do you eat frequently (please make multiple checks if applicable)?

- Vegetable dish [X100a1=0.6 if checked, else 0]
- Meat dish [X100a2=0.4 if checked, else 0]
- Cereal/grain/corn[X100a3=0.2 if checked, else 0]

1. How many times do you eat leftovers a month on average?

- 1-6 [X100b =0.2]
- 7-12 [X100b =0.4]
- 13-18[X100b =0.6]
- 19-24 [X100b =0.8]
- 25-30 [X100b =1.0]
- 31 and more [X100b =1.2]

[X100a= X100a1+ X100a2+ X100a3]

[X100= (X100a + X100b)/2]

**X101: Cottonseed oil (if unchecked in rapid assessment, X101= 0 and skip to X112)**

1. How many months do you eat cottonseed oil a year on average?

- 1-2 [X101 =0.2]
- 3-4[X101 =0.4]
- 5-6[X101 =0.6]
- 7-8 [X101 =0.8]
- 9-10 [X101 =1.0]
- 11-12[X101 =1.2]

**X111: Drinking tea (if unchecked in rapid assessment, X111= 0 and skip to X112)**

1. How many years in your life can you be described as frequent tea drinker?

- 1-2 [X111a =-0.2]
- 3-4[X111a =-0.4]
- 5-6[X111a =-0.6]
- 7-8 [X111a =-0.8]
- 9-10 [X111a =-1.0]
- 11 and more[X111a =-1.2]

1. How many cups of tea you drink a day?

- 1[X111b =-0.3]
- 2[X111b =-0. 6]
- 3[X111b =-0. 9]
- 4 and more[X111b =-1.2]

[X111 =( X111a+ X111b)/2]

**X112: Drinking coffee (if unchecked in rapid assessment, X112= 0 and skip to X113)**

1. How many years in your life can you be described as frequent coffee drinker?

- 1-2 [X112a =0.2]
- 3-4[X112a =0.4]
- 5-6[X112a =0.6]
- 7-8 [X112a =0.8]
- 9-10 [X112a =1.0]
- 11 and more[X112a =1.2]

1. How many cups of coffee you drink a day?

- 1[X112b =0.3]
- 2[X112b =0. 6]
- 3[X112b =0. 9]
- 4 and more[X112b =1.2]

[X112 =( X112a+ X112b)/2]

**X113: Drinking alcohol (if unchecked in rapid assessment, X113= 0 and skip to X114)**

1. How many years in your life can you be described as frequent drinker?

- 1-5 [X113a=0.2]
- 6-10 [X113a =0.4]
- 11-15 [X113a =0.6]
- 16-20 [X113a =0.8]
- 21-25 [X113a =1.0]
- 26 and more [X113a =1.2]

1. How many times did you drink a month?

- 1-6 [X113b =0.2]
- 7-12 [X113b =0.4]
- 13-18[X113b =0.6]
- 19-24 [X113b =0.8]
- 25-30 [X113b =1.0]
- 31 and more [X113b =1.2]

1. How much alcohol did you drink per time?

- 1-10 g[X113c =0]
- 11-30g[X113c=0.3]
- 31-50g[X113c=0.6]
- 51-70g[X113c=0.9]
- 71g and more [x113c=1.2]

1. How many times did you over drink a month?

- 1-6 [X113d =0.2]
- 7-12 [X113d =0.4]
- 13-18[X113d =0.6]
- 19-24 [X113d =0.8]
- 25-30 [X113d =1.0]
- 31 and more [X113d =1.2]

[X113= (X113a+ X113b+ X113c+ X113d)/4]

**X114: Smoker(if unchecked in rapid assessment, X114= 0 and skip to X115)**

1. How old were you when you started smoking?

- 15 and less [X114a=1.2]
- 1 6-20 [X114a =1.0]
- 21-25 [X114a =0.8]
- 26-30 [X114a =0.6]
- 31-35 [X114a =0.4]
- 36 and older [X114a =0.2]

1. How many cigarettes do you smoke a day?

- 1-5 [X114b=0.2]
- 6-10 [X114b =0.4]
- 11-15 [X114b =0.6]
- 16-20 [X114b =0.8]
- 21-25 [X114b =1.0]
- 26 and more [X114b =1.2]

1. How many years have you smoked excluding cessation time?

- 1-5 [X114c=0.2]
- 6-10 [X114c =0.4]
- 11-15 [X114c =0.6]
- 16-20 [X114c =0.8]
- 21-25 [X114c =1.0]
- 26 and more [X114c =1.2]

[X114=( X114a+ X114b+ X114c)/3]

**X115: Sedentary person (if unchecked in rapid assessment, X115= 0 and skip to X116)**

1. How much time you spend on heavy physical activities (such as heavy lifting, digging, logging) a day?

- ____hours [X115a = entered value]

1. How much time you spend on moderate physical activities (such as carrying small items, sweeping, laundry, cooking, housekeeping) a day?

- ____hours [X115b = entered value]

1. How much time you spend sitting a day?

- ____hours [X115c = entered value]

1. How much time you spend sleeping (including siesta, starting from the time lying in bed) a day?

- ____hours [X115d = entered value]

[X115=0.2, 0.4, 0.6, 0.8, 1.0, 1.2 respectively if X115a *17+X115b*8+X115c*4+ X115d*3= 171 and more, 151-170,131-150,111-130, 91-110, 90 and less]

**X116: Night owl (if unchecked in rapid assessment, X116= 0 and skip to X117)**

1. How many times you stay up late a month?

- 1-5 [X116a =0.2]
- 6-10 [X116a =0.4]
- 11-15[X116a =0.6]
- 16-20 [X116a =0.8]
- 21-25[X116a =1.0]
- 26 and more [X116a =1.2]

1. Which season(s) of the year do you often stay up late (please make multiple checks if applicable)?

- Spring [X116b1=0.3 if checked, else 0]
- Summer[X116b2=0.3 if checked, else 0]
- Autumn[X116b3=0.3 if checked, else 0]
- Winter[X116b4=0.3 if checked, else 0]

[X116b = (X116b1+ X116b2+ X116b3+ X116b4)]

[X116=(X116a+X116b)/2]

**X117: Renovation worker/painter (if unchecked in rapid assessment, X117= 0 and skip to X118)**

1. How many years have you been renovation worker or painter?

- 1-5 [X117a=0.2]
- 6-10 [X117a =0.4]
- 11-15 [X117a =0.6]
- 16-20 [X117a =0.8]
- 21-25 [X117a =1.0]
- 26 and more [X117a =1.2]

1. How often did you take protective measures when you were working?

- Not at all [X117b =1.2]
- Occasionally [X117b =0.8]
- Most of the time[X117b =0.4]
- Every time[X117b =0]

[X117=(X117a+X117b)/2]

**X118: Carpenter (if unchecked in rapid assessment, X118= 0 and skip to X119)**

1. How many years have you been carpenter?

- 1-5 [X118a=0.2]
- 6-10 [X118a =0.4]
- 11-15 [X118a =0.6]
- 16-20 [X118a =0.8]
- 21-25 [X118a =1.0]
- 26 and more [X118a =1.2]

1. How often did you take protective measures when you were working?

- Not at all [X118b =1.2]
- Occasionally [X118b =0.8]
- Most of the time[X118b =0.4]
- Every time[X118b =0]

[X118=( X118a + X118b)/2]

**X119: Cooker (if unchecked in rapid assessment, X119= 0 and skip to X120)**

1. How many years have you been cooker?

- 1-5 [X119a=0.2]
- 6-10 [X119a =0.4]
- 11-15 [X119a =0.6]
- 16-20 [X119a =0.8]
- 21-25 [X119a =1.0]
- 26 and more [X119a =1.2]

1. How often did you take protective measures when you were working?

- Not at all [X119b =1.2]
- Occasionally [X119b =0.8]
- Most of the time[X119b =0.4]
- Every time[X119b =0]

[X119=( X119a + X119b)/2]

**X120: Barber (if unchecked in rapid assessment, X120= 0 and skip to X121)**

1. How many years have you been barber?

- 1-5 [X120a=0.2]
- 6-10 [X120a =0.4]
- 11-15 [X120a =0.6]
- 16-20 [X120a =0.8]
- 21-25 [X120a =1.0]
- 26 and more [X120a =1.2]

1. How often did you take protective measures when you were working?

- Not at all [X120b =1.2]
- Occasionally [X120b =0.8]
- Most of the time[X120b =0.4]
- Every time[X120b =0]

[X120=( X120a + X120b)/2]

**X121: Aluminum** **worker (if unchecked in rapid assessment, X121= 0 and skip to X122)**

1. How many years have you been aluminum worker?

- 1-5 [X121a=0.2]
- 6-10 [X121a =0.4]
- 11-15 [X121a =0.6]
- 16-20 [X121a =0.8]
- 21-25 [X121a =1.0]
- 26 and more [X121a =1.2]

1. How often did you take protective measures when you were working?

- Not at all [X121b =1.2]
- Occasionally [X121b =0.8]
- Most of the time[X121b =0.4]
- Every time[X121b =0]

[X121= ( X121a + X121b)/2]

**X122: Welder (if unchecked in rapid assessment, X122= 0 and skip to X123)**

1. How many years have you been welder?

- 1-5 [X122a=0.2]
- 6-10 [X122a =0.4]
- 11-15 [X122a =0.6]
- 16-20 [X122a =0.8]
- 21-25 [X122a =1.0]
- 26 and more [X122a =1.2]

1. How often did you take protective measures when you were working?

- Not at all [X122b =1.2]
- Occasionally [X122b =0.8]
- Most of the time[X122b =0.4]
- Every time[X122b =0]

[X122=(X122a+ X122b)/2]

**X123: Miner (if unchecked in rapid assessment, X123= 0 and skip to X124)**

1. How many years have you been miner?

- 1-5 [X123a=0.2]
- 6-10 [X123a =0.4]
- 11-15 [X123a =0.6]
- 16-20 [X123a =0.8]
- 21-25 [X123a =1.0]
- 26 and more [X123a =1.2]

1. How often did you take protective measures when you were working?

- Not at all [X123b =1.2]
- Occasionally [X123b =0.8]
- Most of the time[X123b =0.4]
- Every time[X123b =0]

[X123=( X123a + X123b)/2]

**X124:** **Chemical** **worker (if unchecked in rapid assessment, X124= 0 and skip to X125)**

1. How many years have you been chemical worker?

- 1-5 [X124a=0.2]
- 6-10 [X124a =0.4]
- 11-15 [X124a =0.6]
- 16-20 [X124a =0.8]
- 21-25 [X124a =1.0]
- 26 and more [X124a =1.2]

1. How often did you take protective measures when you were working?

- Not at all [X124b =1.2]
- Occasionally [X124b =0.8]
- Most of the time[X124b =0.4]
- Every time[X124b =0]

[X124= ( X124a + X124b)/2]

**X125: Boiler worker (if unchecked in rapid assessment, X125= 0 and skip to X126)**

1. How many years have you been boiler worker?

- 1-5 [X125a=0.2]
- 6-10 [X125a =0.4]
- 11-15 [X125a =0.6]
- 16-20 [X125a =0.8]
- 21-25 [X125a =1.0]
- 26 and more [X125a =1.2]

1. How often did you take protective measures when you were working?

- Not at all [X125b =1.2]
- Occasionally [X125b =0.8]
- Most of the time[X125b =0.4]
- Every time[X125b =0]

[X125=( X125a + X125b)/2]

**X126: Asphalt/resin worker (if unchecked in rapid assessment, X126= 0 and skip to X127)**

1. How many years have you been asphalt/resin worker?

- 1-5 [X126a=0.2]
- 6-10 [X126a =0.4]
- 11-15 [X126a =0.6]
- 16-20 [X126a =0.8]
- 21-25 [X126a =1.0]
- 26 and more [X126a =1.2]

1. How often did you take protective measures when you were working?

- Not at all [X126b =1.2]
- Occasionally [X126b =0.8]
- Most of the time[X126b =0.4]
- Every time[X126b =0]

[X126=( X126a + X126b)/2]

**X127: Dye/pigments worker (if unchecked in rapid assessment, X127= 0 and skip to X128)**

1. How many years have you been dye/pigments worker?

- 1-5 [X127a=0.2]
- 6-10 [X127a =0.4]
- 11-15 [X127a =0.6]
- 16-20 [X127a =0.8]
- 21-25 [X127a =1.0]
- 26 and more [X127a =1.2]

1. How often did you take protective measures when you were working?

- Not at all [X127b =1.2]
- Occasionally [X127b =0.8]
- Most of the time[X127b =0.4]
- Every time[X127b =0]

[X127=(X127a+ X127b)/2]

**X128: Rubber/plastic worker (if unchecked in rapid assessment, X128= 0 and skip to X129)**

1. How many years have you been rubber/plastic worker?

- 1-5 [X128a=0.2]
- 6-10 [X128a =0.4]
- 11-15 [X128a =0.6]
- 16-20 [X128a =0.8]
- 21-25 [X128a =1.0]
- 26 and more [X128a =1.2]

1. How often did you take protective measures when you were working?

- Not at all [X128b =1.2]
- Occasionally [X128b =0.8]
- Most of the time[X128b =0.4]
- Every time[X128b =0]

[X128=( X128a+ X128b)/2]

**X129: Gas worker (if unchecked in rapid assessment, X129= 0 and skip to X130)**

1. How many years have you been gas worker?

- 1-5 [X129a=0.2]
- 6-10 [X129a =0.4]
- 11-15 [X129a =0.6]
- 16-20 [X129a =0.8]
- 21-25 [X129a =1.0]
- 26 and more [X129a =1.2]

1. How often did you take protective measures when you were working?

- Not at all [X129b =1.2]
- Occasionally [X129b =0.8]
- Most of the time[X129b =0.4]
- Every time[X129b =0]

[X129=( X129a + X129b)/2]

**X130: X-ray or radiation related worker (if unchecked in rapid assessment, X130= 0 and skip to X131)**

1. How many years have you been X-ray or radiation related worker?

- 1-5 [X130a=0.2]
- 6-10 [X130a =0.4]
- 11-15 [X130a =0.6]
- 16-20 [X130a =0.8]
- 21-25 [X130a =1.0]
- 26 and more [X130a =1.2]

1. How often did you take protective measures when you were working?

- Not at all [X130b =1.2]
- Occasionally [X130b =0.8]
- Most of the time[X130b =0.4]
- Every time[X130b =0.0]

[X130=(X130a + X130b)/2]

**X131: Pesticides (if unchecked in rapid assessment, X131= 0 and skip to X132)**

1. How many years in your lifetime have you been exposed to pesticides?

- 1-5 [X131a=0.2]
- 6-10 [X131a =0.4]
- 11-15 [X131a =0.6]
- 16-20 [X131a =0.8]
- 21-25 [X131a =1.0]
- 26 and more [X131a =1.2]

1. How many months were you exposed to pesticides a year?

- 1-2 [X131b=0.2]
- 3-4[X131b=0.4]
- 5-6[X131b=0.6]
- 7-8[X131b=0.8]
- 9-10[X131b=1.0]
- 11-12[X131b=1.2]

1. How many days were you exposed to pesticides a month?

- 1-5[X131c=0.2]
- 6-10[X131c=0.4]
- 11-15[X131c=0.6]
- 16-20[X131c=0.8]
- 21-25[X131c=1.0]
- 26 and more[X131c=1.2]

1. How often did you take protective measures when you were exposed to pesticides?

- Not at all [X131d =1.2]
- Occasionally [X131d =0.8]
- Most of the time[X131d =0.4]
- Every time[X131d =0.0]

[X131=( X131a+ X131b+ X131c+ X131d)/4]

**X132:** Cooking fumes **(if unchecked in rapid assessment, X132= 0 and skip to X133)**

1. How many years in your lifetime have you been exposed to cooking fumes?

- 1-5 [X132a=0.2]
- 6-10 [X132a =0.4]
- 11-15 [X132a =0.6]
- 16-20 [X132a =0.8]
- 21-25 [X132a =1.0]
- 26 and more [X132a =1.2]

1. How many months were you exposed to cooking fumes a year?

- 1-2 [X132b=0.2]
- 3-4[X132b =0.4]
- 5-6[X132b =0.6]
- 7-8[X132b =0.8]
- 9-10[X132b =1.0]
- 11-12[X132b =1.2]

1. How many days were you exposed to cooking fumes a month?

- 1-5[X132c=0.2]
- 6-10[X132c =0.4]
- 11-15[X132c =0.6]
- 16-20[X132c =0.8]
- 21-25[X132c =1.0]
- 26 and more[X132c =1.2]

1. How often did you take protective measures when you were exposed to cooking fumes?

- Not at all [X132d =1.2]
- Occasionally [X132d =0.8]
- Most of the time[X132d =0.4]
- Every time[X132d =0.0]

[X132= ( X132a+ X132b+ X132c+ X132d)/4]

**X133: Firewood smoke (if unchecked in rapid assessment, X133= 0 and skip to X134)**

1. How many years in your lifetime have you been exposed to firewood smoke?

- 1-5 [X133a=0.2]
- 6-10 [X133a =0.4]
- 11-15 [X133a =0.6]
- 16-20 [X133a =0.8]
- 21-25 [X133a =1.0]
- 26 and more [X133a =1.2]

1. How many months were you exposed to firewood smoke a year?

- 1-2 [X133b=0.2]
- 3-4[X133b =0.4]
- 5-6[X133b =0.6]
- 7-8[X133b =0.8]
- 9-10[X133b =1.0]
- 11-12[X133b =1.2]

1. How many days were you exposed to firewood smoke a month?

- 1-5[X133c=0.2]
- 6-10[X133c =0.4]
- 11-15[X133c =0.6]
- 16-20[X133c =0.8]
- 21-25[X133c =1.0]
- 26 and more[X133c =1.2]

1. How often did you take protective measures when you were exposed to firewood smoke?

- Not at all [X133d =1.2]
- Occasionally [X133d =0.8]
- Most of the time[X133d =0.4]
- Every time[X133d =0.0]

[X133=( X133a + X133b + X133c + X133d)/4]

**X134: Soot** **(if unchecked in rapid assessment, X134= 0 and skip to X135)**

1. How many years in your lifetime have you been exposed to soot?

- 1-5 [X134a=0.2]
- 6-10 [X134a =0.4]
- 11-15 [X134a =0.6]
- 16-20 [X134a =0.8]
- 21-25 [X134a =1.0]
- 26 and more [X134a =1.2]

1. How many months were you exposed to soot a year?

- 1-2 [X134b=0.2]
- 3-4[X134b =0.4]
- 5-6[X134b =0.6]
- 7-8[X134b =0.8]
- 9-10[X134b =1.0]
- 11-12[X134b =1.2]

1. How many days were you exposed to soot a month?

- 1-5[X134c=0.2]
- 6-10[X134c =0.4]
- 11-15[X134c =0.6]
- 16-20[X134c =0.8]
- 21-25[X134c =1.0]
- 26 and more[X134c =1.2]

1. How often did you take protective measures when you were exposed to soot?

- Not at all [X134d =1.2]
- Occasionally [X134d =0.8]
- Most of the time[X134d =0.4]
- Every time[X134d =0.0]

[X134=( X134a + X134b + X134c + X134d)/4]

**X135: Dust or cotton dust (if unchecked in rapid assessment, X135= 0 and skip to X136)**

1. How many years in your lifetime have you been exposed to dust or cotton dust?

- 1-5 [X135a=0.2]
- 6-10 [X135a =0.4]
- 11-15 [X135a =0.6]
- 16-20 [X135a =0.8]
- 21-25 [X135a =1.0]
- 26 and more [X135a =1.2]

1. How many months were you exposed to dust or cotton dust a year?

- 1-2 [X135b=0.2]
- 3-4[X135b =0.4]
- 5-6[X135b =0.6]
- 7-8[X135b =0.8]
- 9-10[X135b =1.0]
- 11-12[X135b =1.2]

1. How many days were you exposed to dust or cotton dust a month?

- 1-5[X135c=0.2]
- 6-10[X135c =0.4]
- 11-15[X135c =0.6]
- 16-20[X135c =0.8]
- 21-25[X135c =1.0]
- 26 and more[X135c =1.2]

1. How often did you take protective measures when you were exposed to dust or cotton dust?

- Not at all [X135d =1.2]
- Occasionally [X135d =0.8]
- Most of the time[X135d =0.4]
- Every time[X135d =0.0]

[X135=( X135a + X135b + X135c + X135d)/4]

**X136: Mosquitocide (if unchecked in rapid assessment, X136= 0 and skip to X137)**

1. How many years in your lifetime have you been exposed to mosquitocide?

- 1-5 [X136a=0.2]
- 6-10 [X136a =0.4]
- 11-15 [X136a =0.6]
- 16-20 [X136a =0.8]
- 21-25 [X136a =1.0]
- 26 and more [X136a =1.2]

1. How many months were you exposed to mosquitocide a year?

- 1-2 [X136b=0.2]
- 3-4[X136b =0.4]
- 5-6[X136b =0.6]
- 7-8[X136b =0.8]
- 9-10[X136b =1.0]
- 11-12[X136b =1.2]

1. How many days were you exposed to mosquitocide a month?

- 1-5[X136c=0.2]
- 6-10[X136c =0.4]
- 11-15[X136c =0.6]
- 16-20[X136c =0.8]
- 21-25[X136c =1.0]
- 26 and more[X136c =1.2]

1. How often did you take protective measures when you were exposed to mosquito or mosquitocide?

- Not at all [X136d =1.2]
- Occasionally [X136d =0.8]
- Most of the time[X136d =0.4]
- Every time[X136d =0.0]

[X136=( X136a + X136b + X136c + X136d)/4]

**X137: Formaldehyde (if unchecked in rapid assessment, X137= 0 and skip to X138)**

1. How many years in your lifetime have you been exposed to formaldehyde?

- 1-5 [X137a=0.2]
- 6-10 [X137a =0.4]
- 11-15 [X137a =0.6]
- 16-20 [X137a =0.8]
- 21-25 [X137a =1.0]
- 26 and more [X137a =1.2]

1. How many months were you exposed to formaldehyde a year?

- 1-2 [X137b=0.2]
- 3-4[X137b =0.4]
- 5-6[X137b =0.6]
- 7-8[X137b =0.8]
- 9-10[X137b =1.0]
- 11-12[X137b =1.2]

1. How many days were you exposed to formaldehyde a month?

- 1-5[X137c=0.2]
- 6-10[X137c =0.4]
- 11-15[X137c =0.6]
- 16-20[X137c =0.8]
- 21-25[X137c =1.0]
- 26 and more[X137c =1.2]

1. How often did you take protective measures when you were exposed to formaldehyde?

- Not at all [X137d =1.2]
- Occasionally [X137d =0.8]
- Most of the time[X137d =0.4]
- Every time[X137d =0.0]

[X137=( X137a + X137b + X133c + X137d)/4]

**X138: Coal tar (if unchecked in rapid assessment, X138= 0 and skip to X139)**

1. How many years in your lifetime have you been exposed to coal tar?

- 1-5 [X138a=0.2]
- 6-10 [X138a =0.4]
- 11-15 [X138a =0.6]
- 16-20 [X138a =0.8]
- 21-25 [X138a =1.0]
- 26 and more [X138a =1.2]

1. How many months were you exposed to coal tar a year?

- 1-2 [X138b=0.2]
- 3-4[X138b =0.4]
- 5-6[X138b =0.6]
- 7-8[X138b =0.8]
- 9-10[X138b =1.0]
- 11-12[X138b =1.2]

1. How many days were you exposed to coal tar a month?

- 1-5[X138c =0.2]
- 6-10[X138c =0.4]
- 11-15[X138c =0.6]
- 16-20[X138c =0.8]
- 21-25[X138c =1.0]
- 26 and more[X133c =1.2]

1. How often did you take protective measures when you were exposed to coal tar?

- Not at all [X138d =1.2]
- Occasionally [X138d =0.8]
- Most of the time[X138d =0.4]
- Every time[X138d =0.0]

[X138=( X138a + X138b + X138c + X138d)/4]

**X139: Chlorophenol (if unchecked in rapid assessment, X139= 0 and skip to X140)**

1. How many years in your lifetime have you been exposed to chlorophenol?

- 1-5 [X139a=0.2]
- 6-10 [X139a =0.4]
- 11-15 [X139a =0.6]
- 16-20 [X139a =0.8]
- 21-25 [X139a =1.0]
- 26 and more [X139a =1.2]

1. How many months were you exposed to chlorophenol a year?

- 1-2 [X139b=0.2]
- 3-4[X139b =0.4]
- 5-6[X139b =0.6]
- 7-8[X139b =0.8]
- 9-10[X139b =1.0]
- 11-12[X139b =1.2]

1. How many days were you exposed to chlorophenol a month?

- 1-5[X139c=0.2]
- 6-10[X139c =0.4]
- 11-15[X139c =0.6]
- 16-20[X139c =0.8]
- 21-25[X139c =1.0]
- 26 and more[X139c =1.2]

1. How often did you take protective measures when you were exposed to chlorophenol?

- Not at all [X139d =1.2]
- Occasionally [X139d =0.8]
- Most of the time[X139d =0.4]
- Every time[X139d =0.0]

[X139=( X139a + X139b + X139c + X139d)/4]

**X140: Arsenic (if unchecked in rapid assessment, X140= 0 and skip to X141)**

1. How many years in your lifetime have you been exposed to arsenic?

- 1-5 [X140a=0.2]
- 6-10 [X140a =0.4]
- 11-15 [X140a =0.6]
- 16-20 [X140a =0.8]
- 21-25 [X140a =1.0]
- 26 and more [X140a =1.2]

1. How many months were you exposed to arsenic a year?

- 1-2 [X140b=0.2]
- 3-4[X140b =0.4]
- 5-6[X140b =0.6]
- 7-8[X140b =0.8]
- 9-10[X140b =1.0]
- 11-12[X140b =1.2]

1. How many days were you exposed to arsenic a month?

- 1-5[X140c=0.2]
- 6-10[X140c =0.4]
- 11-15[X140c =0.6]
- 16-20[X140c =0.8]
- 21-25[X140c =1.0]
- 26 and more[X140c =1.2]

1. How often did you take protective measures when you were exposed to arsenic?

- Not at all [X140d =1.2]
- Occasionally [X140d =0.8]
- Most of the time[X140d =0.4]
- Every time[X140d =0.0]

[X140=( X140a + X140b + X140c + X140d)/4]

**X141: Chrome (if unchecked in rapid assessment, X141= 0 and skip to X142)**

1. How many years in your lifetime have you been exposed to chrome?

- 1-5 [X141a=0.2]
- 6-10 [X141a =0.4]
- 11-15 [X141a =0.6]
- 16-20 [X141a =0.8]
- 21-25 [X141a =1.0]
- 26 and more [X141a =1.2]

1. How many months were you exposed to chrome a year?

- 1-2 [X141b=0.2]
- 3-4[X141b =0.4]
- 5-6[X141b =0.6]
- 7-8[X141b =0.8]
- 9-10[X141b =1.0]
- 11-12[X141b =1.2]

1. How many days were you exposed to chrome a month?

- 1-5[X141c=0.2]
- 6-10[X141c =0.4]
- 11-15[X141c =0.6]
- 16-20[X141c =0.8]
- 21-25[X141c =1.0]
- 26 and more[X141c =1.2]

1. How often did you take protective measures when you were exposed to chrome?

- Not at all [X141d =1.2]
- Occasionally [X141d =0.8]
- Most of the time[X141d =0.4]
- Every time[X141d =0.0]

[X141=( X141a + X141b + X141c + X141d)/4]

**X142: Radon (if unchecked in rapid assessment, X142= 0 and skip to X143)**

1. How many years in your lifetime have you been exposed to radon?

- 1-5 [X142a=0.2]
- 6-10 [X142a =0.4]
- 11-15 [X142a =0.6]
- 16-20 [X142a =0.8]
- 21-25 [X142a =1.0]
- 26 and more [X142a =1.2]

1. How many months were you exposed to radon a year?

- 1-2 [X142b=0.2]
- 3-4[X142b =0.4]
- 5-6[X142b =0.6]
- 7-8[X142b =0.8]
- 9-10[X142b =1.0]
- 11-12[X142b =1.2]

1. How many days were you exposed to radon a month?

- 1-5[X142c=0.2]
- 6-10[X142c =0.4]
- 11-15[X142c =0.6]
- 16-20[X142c =0.8]
- 21-25[X142c =1.0]
- 26 and more[X142c =1.2]

1. How often did you take protective measures when you were exposed to radon?

- Not at all [X142d =1.2]
- Occasionally [X142d =0.8]
- Most of the time[X142d =0.4]
- Every time[X142d =0.0]

[X142=( X142a + X142b + X142c + X142d)/4]

**X143: Asbestos (if unchecked in rapid assessment, X143= 0 and skip to X144)**

1. How many years in your lifetime have you been exposed to asbestos?

- 1-5 [X143a=0.2]
- 6-10 [X143a =0.4]
- 11-15 [X143a =0.6]
- 16-20 [X143a =0.8]
- 21-25 [X143a =1.0]
- 26 and more [X143a =1.2]

1. How many months were you exposed to asbestos a year?

- 1-2 [X143b=0.2]
- 3-4[X143b =0.4]
- 5-6[X143b =0.6]
- 7-8[X143b =0.8]
- 9-10[X143b =1.0]
- 11-12[X143b =1.2]

1. How many days were you exposed to asbestos a month?

- 1-5[X143c=0.2]
- 6-10[X143c =0.4]
- 11-15[X143c =0.6]
- 16-20[X143c =0.8]
- 21-25[X143c =1.0]
- 26 and more[X143c =1.2]

1. How often did you take protective measures when you were exposed to asbestos?

- Not at all [X143d =1.2]
- Occasionally [X143d =0.8]
- Most of the time[X143d =0.4]
- Every time[X143d =0.0]

[X143= ( X143a + X143b + X143c + X143d)/4]

**X144: Benzene (if unchecked in rapid assessment, X144= 0 and skip to X145)**

1. How many years in your lifetime have you been exposed to benzene?

- 1-5 [X144a=0.2]
- 6-10 [X144a =0.4]
- 11-15 [X144a =0.6]
- 16-20 [X144a =0.8]
- 21-25 [X144a =1.0]
- 26 and more [X144a =1.2]

1. How many months were you exposed to benzene a year?

- 1-2 [X144b=0.2]
- 3-4[X144b =0.4]
- 5-6[X144b =0.6]
- 7-8[X144b =0.8]
- 9-10[X144b =1.0]
- 11-12[X144b =1.2]

1. How many days were you exposed to benzene a month?

- 1-5[X144c=0.2]
- 6-10[X144c =0.4]
- 11-15[X144c =0.6]
- 16-20[X144c =0.8]
- 21-25[X144c =1.0]
- 26 and more[X144c =1.2]

1. How often did you take protective measures when you were exposed to benzene?

- Not at all [X144d =1.2]
- Occasionally [X144d =0.8]
- Most of the time[X144d =0.4]
- Every time[X144d =0.0]

[X144= (X144a + X144b + X144c + X144d)/4]

**X146: House located near polluting factories (if unchecked in rapid assessment, X146= 0 and skip to X147)**

1. How many years have you lived in the house near polluting factories?

- 1-5 [X146=0.2]
- 6-10 [X146=0.4]
- 11-15 [X146=0.6]
- 16-20 [X146=0.8]
- 21-25 [X146=1.0]
- 26 and more [X146=1.2]

**X147: House using open and unprocessed water sources (if unchecked in rapid assessment, X147= 0 and skip to X148)**

1. How many years in your lifetime have you lived in the house using open and unprocessed water sources?

- 1-5 [X147=0.2]
- 6-10 [X147=0.4]
- 11-15 [X147=0.6]
- 16-20 [X147=0.8]
- 21-25 [X147=1.0]
- 26 and more [X147=1.2]

**X148: Death of beloved/family member(s) (if unchecked in rapid assessment, X148= 0 and skip to X149)**

1. To what extent had the loss of your beloved/family member(s) affected you?

- Mildly [X148a=0.3]
- Moderately [X148a=0.6]
- Severely[X148a=0.9]
- Extremely [X148a=1.2]

1. How long had the loss of your beloved/family member(s) affected you?

- Less than 1 month [X148b = 0.2]
- 2-3 months[X148b = 0.4]
- 4-6 months[X148b = 0.6]
- 7-12 months[X148b = 0.8]
- 2-3 years[X148b = 1.0]
- 4 years and more[X148b = 1.2]

[X148=(X148a+X148b)/2]

**X149: Major disease/ injury of beloved/family member(s) (if unchecked in rapid assessment, X149= 0 and skip to X150)**

1. To what extent had the disease/injury of your beloved/family member(s) affected you?

- Mildly [X149a=0.3]
- Moderately [X149a =0.6]
- Severely[X149a =0.9]
- Extremely [X149a =1.2]

1. How long had the disease/injury of your beloved/family member(s) affected you?

- Less than 1 month [X149b = 0.2]
- 2-3 months[X149b = 0.4]
- 4-6 months[X149b = 0.6]
- 7-12 months[X149b = 0.8]
- 2-3 years[X149b = 1.0]
- 4 years and more[X149b = 1.2]

[X149=(X149a+X149b)/2]

**X150: Major disease/ injury of self (if unchecked in rapid assessment, X150= 0 and skip to X151)**

1. To what extent had your disease/injury affected you?

- Mildly [X150a=0.3]
- Moderately [X150a =0.6]
- Severely[X150a =0.9]
- Extremely [X150a =1.2]

1. How long had your disease/injury affected you?

- Less than 1 month [X150b = 0.2]
- 2-3 months[X150b = 0.4]
- 4-6 months[X150b = 0.6]
- 7-12 months[X150b = 0.8]
- 2-3 years[X150b = 1.0]
- 4 years and more [X150b = 1.2]

[X150=(X150a+ X150b)/2]

**X151: Major property damage (if unchecked in rapid assessment, X151= 0 and skip to X152)**

1. To what extent had your major property damage affected you?

- Mildly [X151a=0.3]
- Moderately [X151a =0.6]
- Severely[X151a =0.9]
- Extremely [X151a =1.2]

1. How long had your major property damage affected you?

- Less than 1 month [X151b = 0.2]
- 2-3 months[X151b = 0.4]
- 4-6 months[X151b = 0.6]
- 7-12 months[X151b = 0.8]
- 2-3 years[X151b = 1.0]
- 4 years and more [X151b = 1.2]

[X151=(X151a+ X151b)/2]

**X152: Long -term conflict/dispute with neighbor(s) (if unchecked in rapid assessment, X152= 0 and skip to X153)**

1. To what extent had your long-term conflict/dispute with neighbor(s) affected you?

- Mildly [X152a=0.3]
- Moderately [X152a =0.6]
- Severely[X152a =0.9]
- Extremely [X152a =1.2]

1. How long had your long-term conflict/dispute with neighbor(s) affected you?

- Less than 1 month [X152b = 0.2]
- 2-3 months[X152b = 0.4]
- 4-6 months[X152b = 0.6]
- 7-12 months[X152b = 0.8]
- 2-3 years[X152b = 1.0]
- 4 years and more [X152b = 1.2]

[X152=( X152a + X152b)/2]

**X153: Long -term conflict/dispute with colleague(s) (if unchecked in rapid assessment, X153= 0 and skip to X154)**

1. To what extent had your long-term conflict/dispute with colleague(s) affected you?

- Mildly [X153a=0.3]
- Moderately [X153a =0.6]
- Severely[X153a =0.9]
- Extremely [X153a =1.2]

1. How long had your long-term conflict/dispute with colleague (s) affected you?

- Less than 1 month [X153b = 0.2]
- 2-3 months[X153b = 0.4]
- 4-6 months[X153b = 0.6]
- 7-12 months[X153b = 0.8]
- 2-3 years[X153b = 1.0]
- 4 years and more[X153b = 1.2]

[X153=( X153a + X153b)/2]

**X154: Breakup/discord with spouse or boy/girl-friend (if unchecked in rapid assessment, X154= 0 and skip to X155)**

1. To what extent had your breakup/discord with spouse or boy/girl-friend affected you?

- Mildly [X154a=0.3]
- Moderately [X154a =0.6]
- Severely[X154a =0.9]
- Extremely [X154a =1.2]

1. How long had your breakup/discord with spouse or boy/girl-friend affected you?

- Less than 1 month [X154b = 0.2]
- 2-3 months[X154b = 0.4]
- 4-6 months[X154b = 0.6]
- 7-12 months[X154b = 0.8]
- 2-3 years[X154b = 1.0]
- 4 years and more[X154b = 1.2]

[X154=( X154a + X154b)/2]

**X155: Highly stressful/intensive work (if unchecked in rapid assessment, X155= 0 and skip to X156)**

1. To what extent had your highly stressful/intensive work affected you?

- Mildly [X155a=0.3]
- Moderately [X155a =0.6]
- Severely[X155a =0.9]
- Extremely [X155a =1.2]

1. How long had your highly stressful/intensive work affected you?

- Less than 1 month [X155b = 0.2]
- 2-3 months[X155b = 0.4]
- 4-6 months[X155b = 0.6]
- 7-12 months[X155b = 0.8]
- 2-3 years[X155b = 1.0]
- 4 years and more[X155b = 1.2]

[X155=( X155a + X155b)/2]

**X156: Natural/man-caused disaster(s) (if unchecked in rapid assessment, X156= 0 and skip to X157)**

1. To what extent had your natural/man-caused disaster(s) affected you?

- Mildly [X156a=0.3]
- Moderately [X156a =0.6]
- Severely[X156a =0.9]
- Extremely [X156a =1.2]

1. How long had your natural/man-caused disaster(s) affected you?

- Less than 1 month [X156b= 0.2]
- 2-3 months[X156b = 0.4]
- 4-6 months[X156b = 0.6]
- 7-12 months[X156b = 0.8]
- 2-3 years[X156b = 1.0]
- 4 years and more[X156b = 1.2]

[X156= ( X156a + X156b)/2]

**X157: Litigation(s) (if unchecked in rapid assessment, X157= 0 and end detailed risk assessment)**

1. To what extent had your litigation (s) affected you?

- Mildly [X157a=0.3]
- Moderately [X157a =0.6]
- Severely[X157a =0.9]
- Extremely [X157a =1.2]

1. How long had your litigation (s) affected you?

- Less than 1 month [X157b= 0.2]
- 2-3 months[X157b = 0.4]
- 4-6 months[X157b = 0.6]
- 7-12 months[X157b = 0.8]
- 2-3 years[X157b = 1.0]
- 4 years and more[X157b = 1.2]

[X157= ( X157a + X157b)/2]

[Note: The values of all the remaining variables which are not included in the detailed risk assessment remain the same as assigned during the rapid risk assessment]

**C) Calculation of rapid and detailed risk assessment scores**

Calculation of both the rapid and detailed risk assessment scores utilizes the following formulae:

a) $R=\sum_{k=1}^{9} P_{k}R_{k}$;

b) $R_{k}=\sum_{i=1}^{n} W_{ki}X_{i}$.

Where:

k ranges from 1 to 9 standing for the nine most common cancers in rural China respectively including stomach cancer, esophagus cancer, trachea/bronchus/lung cancer, liver cancer, colon/rectum cancer, breast cancer, cervix cancer, pancreas cancer, and nasopharynx cancer;

*P_k_* represents gender and age-specific incidence rate of cancer *k* in rural China derived from the latest Chinese Cancer Registry Annual Report 2012;

*R_k_* stands for the risk score of cancer *k* for a specific individual farmer under concern;

*n* denotes the number of risk factors included in rapid and detailed (*n*=164) risk assessment;

*X_i_* is the Liker scale of the risk factor *X_i_* generated via the rapid/detailed assessment using the questionnaires and value systems described in the above section A and B;

*W_ki_* refers to the pooled odds ratio of cancer *k* for risk factor *i* derived through systematic review and meta-analysis of published researches on the same odds ratios among farmers in China;

*R* defines the total risk score of the specific farmer under concern for developing any of the 9-cancers.
